# Quantum Chemical Investigation of the Dimroth Rearrangement of 1‐(5‐Imino‐4,5‐dihydro‐1H‐1,2,3‐triazol‐1‐yl)anthracene‐9,10‐dione

**DOI:** 10.1002/open.70330

**Published:** 2026-10-28

**Authors:** Dmytro Melnyk, Vasyl Shupeniuk, Tetyana Taras, Volodymyr Kotsyubynsky

**Affiliations:** ^1^ Department of Chemistry, Pharmaceutical Analysis and Postgraduate Education Ivano‐Frankivsk National Medical University Ivano‐Frankivsk Ukraine; ^2^ Department of Environmental Chemistry and Chemistry Education Vasyl Stefanyk Carpathian National University Ivano‐Frankivsk Ukraine; ^3^ Department of Applied Physics and Materials Science Vasyl Stefanyk Carpathian National University Ivano‐Frankivsk Ukraine

**Keywords:** anthracenedion, density functional calculations, heterocycles, rearrangement, triazole

## Abstract

This work is devoted to quantum chemical and experimental studies of the Dimroth rearrangement mechanism of 1‐(5‐imino‐4,5‐dihydro‐1H‐1,2,3‐triazol‐1‐yl)anthracene‐9,10‐dione. The study addresses how the bulky *N‐*anthraquinonyl substituent affects the individual steps of the Dimroth rearrangement. The energy profile of the Dimroth rearrangement was calculated using density functional theory with the B3LYP approximation and the 6‐311++G(d,p) basis set. It was found that proton solvents (in particular, ethanol) play a critical role in hydrogen transfer via the hydrogen‐bond network, thereby ensuring the conversion of the initial triazene into a more stable cyclic 5‐aminotriazole. Further transformations involving ring opening and rotation do not have significant energy barriers. The final product is energetically most favorable, so the reaction proceeds easily in ethanol solution. The work demonstrates the convenience and efficiency of using the Dimroth rearrangement for the synthesis of new anthracenedione‐containing heterocycles. It provides an explanation of the mechanism of this rearrangement based on quantum‐chemical calculations for anthracene‐9,10‐dione derivatives.

## Graphical abstract

Density functional theory (DFT) reveals how proton transfer involving ethanol leads to a Dimroth rearrangement of an anthraquinone derivative. The calculated energy profile identifies key intermediate compounds and transition states, showing that solvent‐mediated hydrogen‐bonding networks promote cyclization, while ring opening and ring closure occur with low activation barriers, leading to the formation of the thermodynamically favorable product – a triazole.

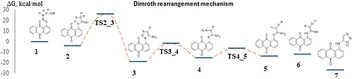

## 1 Introduction

Anthracenediones (anthraquinones) are a class of compounds that form the basis of several drugs with a wide range Anthracene‐9,10‐diones (anthraquinones) and are a well‐established scaffold in medicinal chemistry, underlying compounds with laxative [1], anti‐inflammatory and antitumor [2], antiviral [3], and antimicrobial and antifungal [4] activity, which is largely attributed to the electron‐accepting anthraquinone system capable of forming charge‐transfer complexes with DNA bases [5]. Amino‐substituted anthracenediones are also used as dyes and analytical reagents [6], and new anthraquinone‐based antitumor agents and regulators of reactive oxygen species continue to be reported [7, 8]. The anthraquinone core readily undergoes electrophilic and nucleophilic substitution [9], and the introduction of a triazene group gives access to heterocyclization reactions [10], which makes anthraquinone triazenes convenient precursors of anthracenedione‐containing heterocycles.

In our synthesis of such heterocycles, 1,2,3‐triazoles were formed via the Dimroth rearrangement. The mechanism of this rearrangement in 1,2,3‐triazoles has been studied by density functional theory (DFT): Feng et al. [11] calculated the energy profiles of the ring‐opening, rotation, and ring‐closure stages, identified the open‐chain tautomer as the key intermediate, and located the corresponding transition states. Related DFT studies addressed thermal rearrangements of fused triazole systems and competing reaction pathways [12, 13]. However, these studies deal with small model triazoles or energetic materials. The influence of a bulky, electron‐withdrawing *N‐*anthraquinonyl substituent, whose *peri*‐carbonyl group can form hydrogen bonds with the solvent and sterically shield the adjacent nitrogen atoms, on the individual steps of the rearrangement has not been examined.

Therefore, the aim of this work was to establish, by DFT calculations at the B3LYP/6‐311++G(d,p) level, the energy profile of the cyclization of 1‐(anthraquinonyl)triazene and the subsequent Dimroth rearrangement of 1‐(5‐imino‐4,5‐dihydro‐1H‐1,2,3‐triazol‐1‐yl)anthracene‐9,10‐dione, with particular attention to the role of the protic solvent (ethanol) in the proton‐transfer steps.

## 2 Results and Discussion

### 2.1 Chemistry and Dimroth Rearrangement

Scheme 1 illustrates the probable mechanism of conversion of triazene **1** to triazole **7**. Triazene **1** exists as a mixture of tautomers (AQN = NNHCH_2_CN ↔ AQNHN = NCH_2_CN) in solution at room temperature. The initial cyclization occurs via an intramolecular nucleophilic attack by the cyano group, forming 5‐aminotriazole **3**. The Dimroth rearrangement of derivative **3**, catalyzed by proton solvents, leads to the formation of triazole **7** without the participation of a catalyst, like in the literature for benzene‐containing 5‐aminotriazoles [14].

**SCHEME 1:**
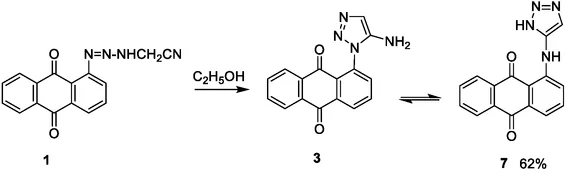
Cyclization of 2‐(3‐(9,10‐dioxo‐9,10‐dihydroanthracen‐1‐yl)triaz‐2‐en‐1‐yl)acetonitrile **1** and Dimroth rearrangement into 1‐((1H‐1,2,3‐triazol‐5‐yl)amino)anthracene‐9,10‐dione **3.**

### 2.2 Structure and Rearrangement Analysis by the Method of DFT Calculation

Modeling the Dimroth cyclization and rearrangement process using quantum‐chemical calculations involves Scheme 2, which exhibits features arising from the peculiarities of the anthraquinone structure compared with other systems for which similar calculations were performed [11, 12, 13, 14, 15].

**SCHEME 2:**
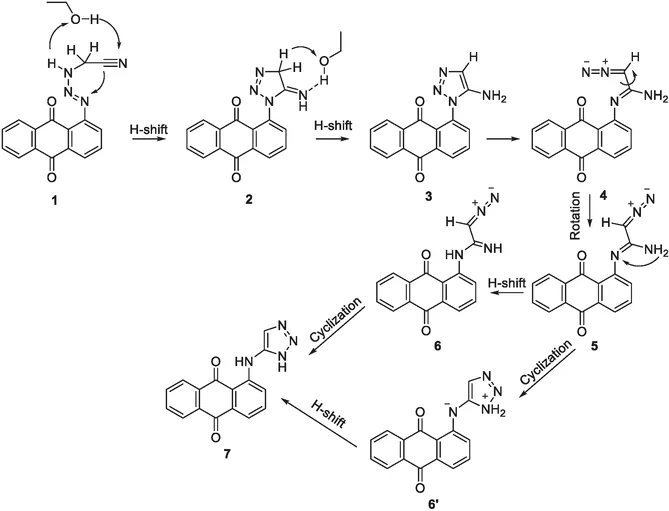
Mechanism of cyclization of triazen **1** and Dimroth rearrangement into triazol **7.**

As can be seen from the calculated energies of all intermediate products, the formed triazene **1**, as a product of the interaction of diazoanthraquinone with aminoacetonitrile, is the energetically least favorable product. Therefore, it is converted to the intermediate cyclic form **2**. This conversion is accompanied by hydrogen transfer. A direct proton transfer between the two carbon atoms is unlikely to occur. Instead, it most likely proceeds via solvent molecules, with ethanol acting as a proton relay in our case. Specifically, the proton may be transferred between the functional groups through a hydrogen‐bonded network involving one or, more likely, two ethanol molecules (see Figure 1). The intermediate form (formed after hydrogen transfer) can have many configurations, and optimizing the geometry of those that allow the cycle to close leads directly to the cyclic 5‐iminodihydrotriazole **2**. Therefore, it can be concluded that after proton transfer, the cyclization process occurs immediately, as a more stable product (Δ*G*1 – Δ*G*2 = 3.83 kcal/mol) is formed, and there are no significant energy barriers to overcome in reducing the energy (see Figure 2). Proton transfer from the methylene group via ethanol is less likely because these hydrogen atoms are less prone to forming a hydrogen bond with ethanol and are located at approximately the same distance from the nitrogen atom (3.12 Å from the methylene hydrogen atom and 3.68 Å from the amino group hydrogen atom).

**FIGURE 1:**
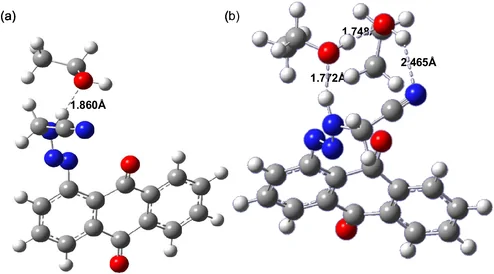
Possibility of forming hydrogen bonds between triazene **1** and one (a) or two (b) alcohol molecules during hydrogen ion transfer.

**FIGURE 2:**
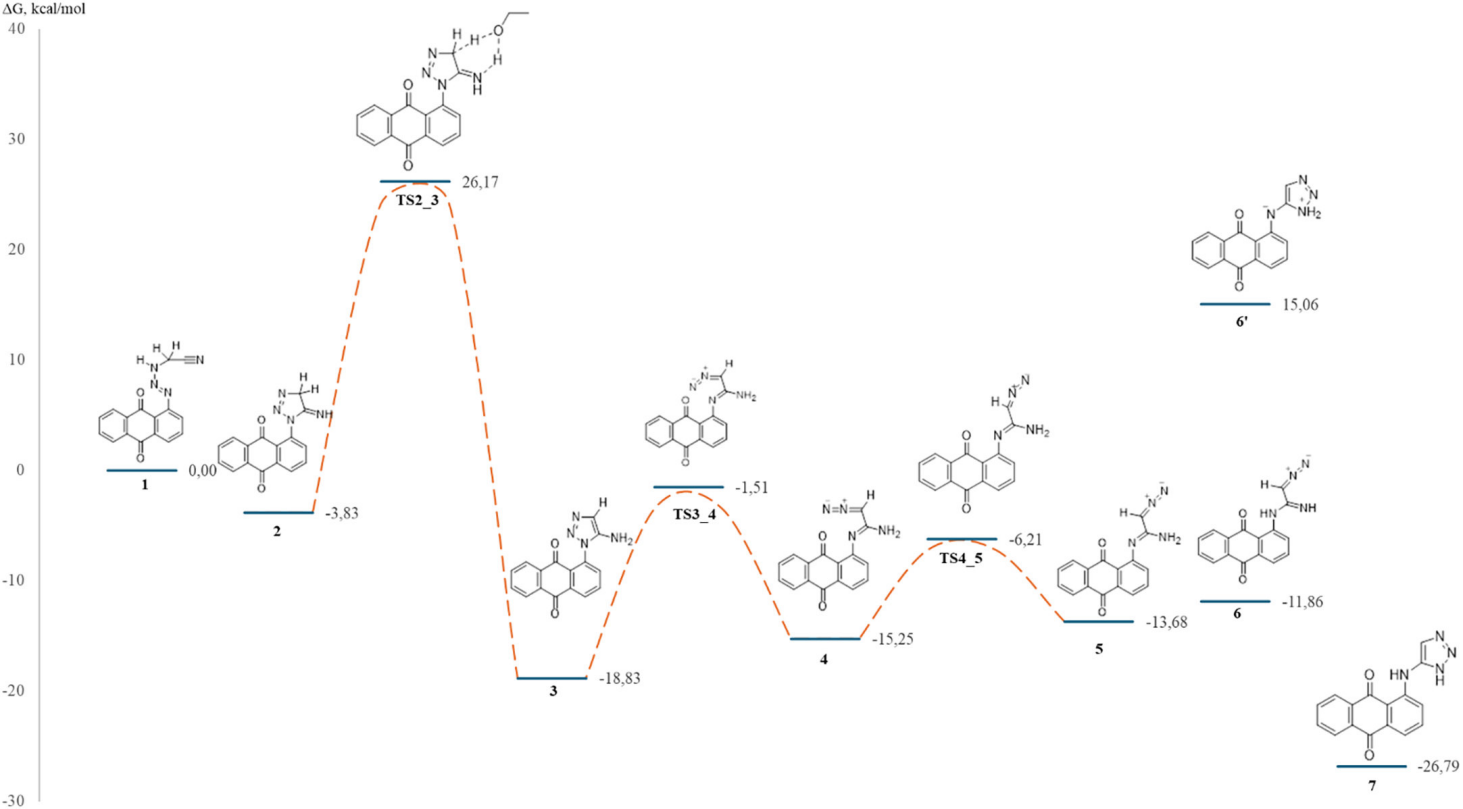
Energy diagram of the conversion of triazene **1** to aminotriazole **7** involving one explicit ethanol molecule, calculated using the IEFPCM model.

The next step is the conversion of 5‐imino dihydrotriazole **2** to 5‐aminotriazole **3**, which can also occur with the participation of solvent molecules. An alcohol molecule can form a hydrogen bond with the nitrogen of the imino group. In the transition state, the alcohol’s hydrogen atom approaches the amine’s nitrogen atom. Accordingly, one of the hydrogen atoms of the ring approaches the oxygen atom of the solvent. The transition state has an energy of 29.99 kcal/mol higher than that of 5‐imino dihydrotriazole **2** (see Figure 3). When checking the vibrations of the transition state, only one imaginary vibration frequency was observed, which corresponds to the displacement of two hydrogen atoms, which means that the transition state is modeled correctly (Figure 2). The formed 5‐aminotriazole **3** with an ethanol molecule bound by a hydrogen bond has an energy of 15.00 kcal/mol lower than that of 5‐imino dihydrotriazole **2**, which suggests that the formed aromatic system is much more energetically favorable.

**FIGURE 3:**
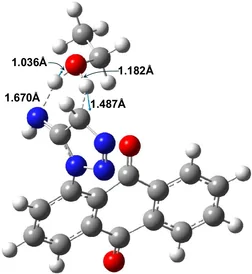
Structure of the transition state (**TS2_3**) involving an ethanol molecule with displacement vectors of imaginary frequency.

The next stage in the Dimroth rearrangement is the cleavage of the ring, as described in the literature, which may occur under the action of a proton from a solvent molecule [11], water [13], or the main solvent on a nitrogen atom bonded to the anthraquinone ring [15]. However, it was not possible to simulate such an interaction due to steric hindrance on one side of the ketone group, which makes it easier for the ketone group to form a hydrogen bond with a solvent proton. On the other hand, the hydrogen atom of the aromatic system creates steric hindrance to the approach of the proton to the nitrogen atom. Therefore, the only possible option is to break the bond without solvent interaction. The Gibbs energy of this transition state (∆*G*_**TS3_4**_) is 17.32 kcal/mol greater than the Gibbs energy of structure **3**, so such a break can occur without significant energy expenditure. The distance between nitrogen atoms increases to 2.14 Å, and the angle N═N─C is 131° (see Figure 4). Due to the low energy barrier, there is no need for solvent initiation for this stage.

**FIGURE 4:**
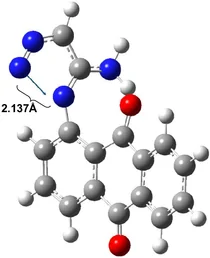
Structure of transition state (**TS3_4**) with displacement vectors of imaginary frequency.

Rotation around the C─C bond, leading to the formation of form **5**, does not require a significant amount of energy (see Figure 2). The Gibbs energy difference between form **4** and **TS4_5** is only 9.04 kcal/mol. The intermediate compound **5** has a Gibbs energy nearly the same as that of **4**, differing by only 1.57 kcal/mol. In the transition state, the rotating group becomes practically perpendicular (see Figure 5), and the torsion angle N═C─C═N is 89.4°. Without steric hindrance, such rotation occurs readily with little energy penalty.

**FIGURE 5:**
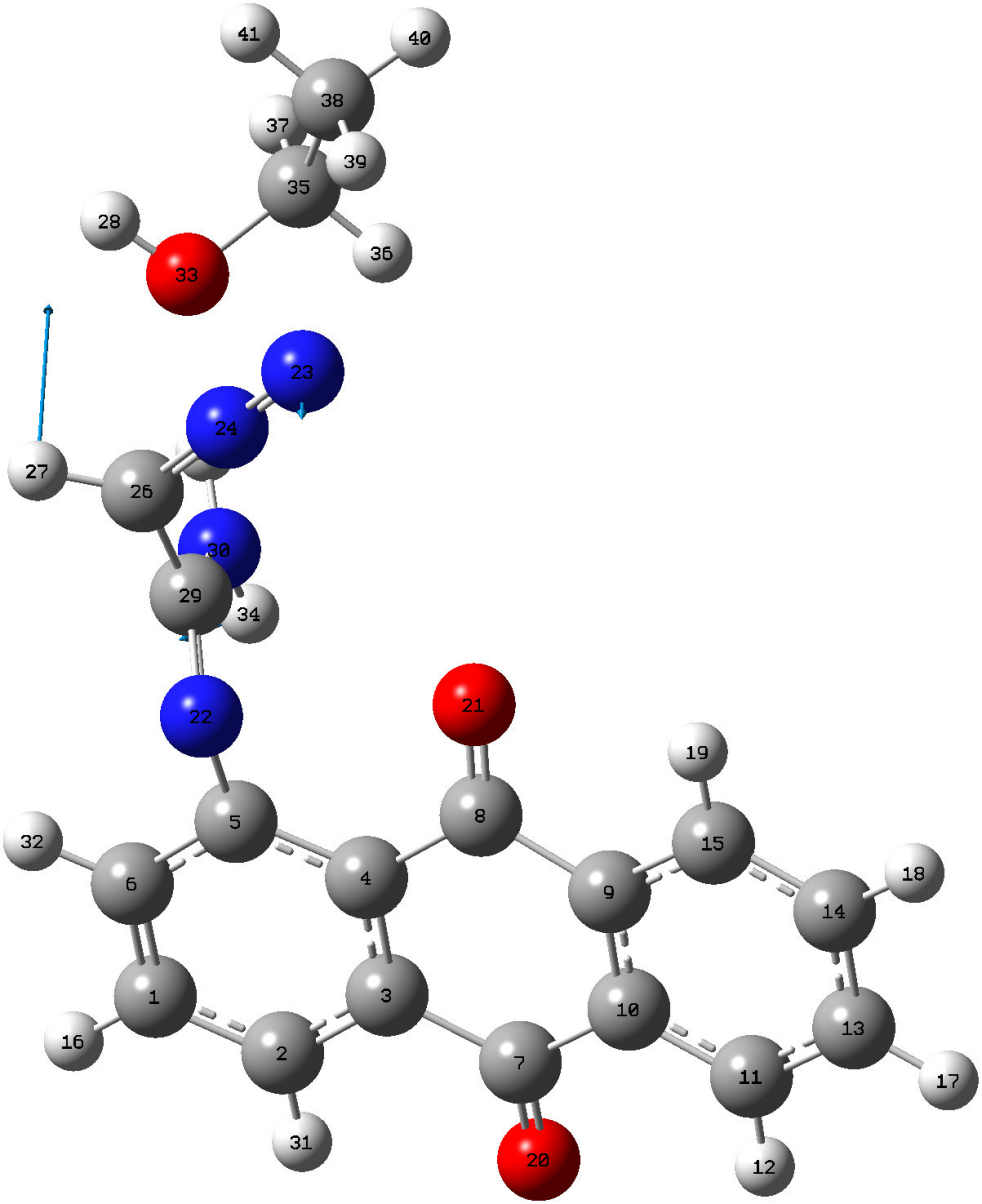
Structure of transition state (**TS4_5**) with displacement vectors of imaginary frequency.

The conversion of intermediate compound **5** into the final product **7** can occur via two possible mechanisms. One of these is a tautomeric transformation of intermediate compound **5** into intermediate compound **6**, followed by ring closure. The other option is ring closure to form compound **6′**, followed by a tautomeric transformation. It was not possible to model all the subsequent transition states in the IEFPCM that contain an ethanol molecule. Most likely, this transformation occurs via compound **6**, which has an energy 26.92 kcal/mol lower than that of the intermediate **6′** (see Figure 2). In the final compound 7, a hydrogen atom forms a hydrogen bond with the oxygen of the carbonyl group, which further stabilizes the product. This results in the formation of the much more stable 1‐((1H‐1,2,3‐triazol‐5‐yl)amino)anthracene‐9,10‐dione **7**, whose energy is 26.79 kcal/mol lower than that of the initial addition product.

### 2.3 X‐Ray Crystallography

The calculated angles of incidence and diffraction of electromagnetic waves relative to the Bragg plane indicate the presence of triazole groups and an anthraquinone structure. The triazole group tends to compact the structure due to high *Ɵ* values (see Table 1). The anthraquinone under study has small or loosely packed molecular structures.

**TABLE 1:** X‐ray powder diffraction of 1‐((1H‐1,2,3‐triazol‐5‐yl)amino)anthracene‐9,10‐dione 7.

Structure	*P* (2‍‐‍theta)	*P* (*I*/*T*_o_)	*I* scale	*I*/*I*_c_	*F*_o_ *M*
Triazole (N─N─N─N)	0.647	0.0523	0.7117	1.6	0.675
Anthraquinone	0.485	0.6175	0.5491	—	0.738

## 3 Conclusion

The synthesis of triazene 1 from the anthraquinone nucleus and its conversion to triazole 7 constitute a convenient route to new anthraquinone derivatives. Quantum‐chemical calculations of this process confirm the need for a protonic solvent, such as ethanol. The calculated energies of intermediate substances, some transition states, and reaction products indicate that, in general, cycle closure and subsequent rearrangement result in a more stable product, and the activation energies of the main stages have relatively low values. X‐ray structural analysis confirmed the structure of the compound formed during the transformation.

## 4 Experimental Section

### 4.1 Materials and Methods

Melting points were measured in open capillary tubes on an IA 9200 Electrothermal melting point apparatus (Bibby Scientific Limited, Stone, UK) and are uncorrected. Elemental analyses (C, H, and N) were performed using the FlashSmart CHNS/O analyzer (Thermo Scientific, Waltham, MA, USA) and were within ±0.4% of the theoretical values. The 400 MHz‐^1^H and 100 MHz‐^13^C spectra were recorded on Varian Mercury 400 (400 MHz) spectrometer (Varian Inc., Paulo Alto, CA, USA). Chemical shifts (*δ*) are quoted in ppm and coupling constants (*J*) are reported in Hz. LC–MS spectra were obtained on Agilent 1260 Infinity II with a single‐quadrupole mass‐selective detector Agilent 6125 (Agilent Technologies, Santa Clara, CA, USA). The reaction mixture was monitored by thin‐layer chromatography (TLC) on commercial glass‐backed plates (DC‐Fertigfolien ALUGRAM Xtra SIL G/UV254, Germany) with eluents of different compositions. IR spectra were recorded on a spectrophotometer, Specord M‐80 (Carl Zeiss Industrielle Messtechnik GmbH, Jena, Germany), in KBr tablets. X‐ray diffraction (XRD) analysis was conducted using the ADX‐2500 XRD system. The triazole sample diffraction pattern was derived via a stepwise scanning process, advancing at a rate of 1º per minute. This procedure encompassed an angular span from 10º to 80º, capturing the diffraction pattern comprehensively. Commercially available solvents and reagents were used without further purification.

### 4.2 General Procedure

#### 4.2.1 General Procedure for the Synthesis of Triazole

Cyanomethyltriazene **1** (0.005 mol) was refluxed in absolute ethanol (50–100 mL) for 1 h. The reaction mixture was evaporated to dryness under reduced pressure, and the resulting residue was purified and analyzed.

### 4.3 Physical and Spectral Data

#### 4.3.1 2‐anthracenedione‐1,2,3‐triazole (7)

Yellow solid, mp >300 °C; yield 62%. ^1^H‐NMR (DMSO‐d_6_, *δ* (ppm)): *δ*6.50 (d, 1H, CH), 7.30–8.20 (m, 7H, H_ar_), 9.30–9.70 (br s, 1H, NH), 14.50–14.80 (br s, 1H, NH). ^13^C NMR (100 MHz, DMSO‐d_6_, *δ* (ppm)): 182.3, 181.7 (C═O), 148.5, 145.2 (C‐triazole), 134.8, 133.5, 132.1, 131.4, 129.8, 128.7, 127.9, 126.5, 126.0, 125.8, 125.5, 124.9 (Ar─C). Anal. calcd. for C_16_H_11_N_4_O_2_ (%): C, 65.97; H, 3.80; N, 19.23. Found (%): C, 65.85; H, 3.92; N, 19.10. IR (KBr, cm^−1^): 1630 (C═O), 1640 (C═N), 3150 (NH), 3550 (triazole ring). MS (*m*/*z*): 436 [M + H]^+^, X‐ray powder diffraction, the sharp diffraction peaks are detected at 2*θ* values of 13.21°, 26.19°, and 46.51° corresponding to triazole.

### 4.4 Quantum Chemical Calculation

All quantum‐chemical calculations were performed using the Gaussian 09 program [16], while molecular structures and vibrational modes were visualized using GaussView 5.0.8. The geometries of all stationary points, including compounds and intermediates 1–7, as well as all transition states, were fully optimized without symmetry constraints using DFT at the B3LYP level, combined with the 6‐311++G(d,p) basis set, with a single ethanol molecule included in the IEFPCM solvent force field model. This basis set, featuring triple *ζ*‐splitting of valence, supplemented by polarization and diffusion functions, provides a reliable description of the electronic structure, particularly for systems involving charge redistribution along reaction pathways, while minimizing the influence of the solvent. Previous studies have confirmed the suitability of the B3LYP functional as a reliable and accurate approach for a range of reactions [17, 18, 19]. Vibrational frequency calculations were carried out at the same theoretical level to confirm the nature of each stationary point. The absence of imaginary frequencies confirmed all minima, while each transition state exhibited a single imaginary frequency corresponding to the reaction coordinate. The associated normal mode was carefully analyzed to ensure that it describes the expected bond‐forming and bond‐breaking processes. To further validate the connectivity of transition states with the corresponding reactants and products, intrinsic reaction coordinate (IRC) calculations were performed at the same level of theory. The IRC paths were traced in both forward and reverse directions, confirming that each transition state smoothly connects the appropriate minima on the potential energy surface. The chosen level of theory provides a reasonable balance between computational cost.

## Funding

This work was supported by Grant of the President of Ukraine for Young Scientists (Grant 130/2025‐rp).

## Conflicts of Interest

The authors declare no conflicts of interest.

## Supporting information

Supplementary Material

## Data Availability

The data that support the findings of this study are available from the corresponding author upon reasonable request.

## References

[1] N. Lombardi , A. Bettiol , G. Crescioli , et al., “Association Between Anthraquinone Laxatives and Colorectal Cancer: Protocol for a Systematic Review and Meta‐Analysis,” Systematic Reviews 9 (2020): 19, 10.1186/s13643-020-1280-5. PMC6979293 31980030

[2] I. H. Gecibesler , F. Disli , S. Bayindir , et al., “The Isolation of Secondary Metabolites From, *Rheum ribes*, L. and the Synthesis of New Semi‐Synthetic Anthraquinones: Isolation, Synthesis and Biological Activity,” Food Chemistry 342 (2021): 128378, 10.1016/j.foodchem.2020.128378. 33508903

[3] A. Lozynskyi , O. Sabadakh , E. Luchkevich , et al., “The Application of Anthraquinone‐Based Triazenes as Equivalents of Diazonium Salts in Reaction With Methylene Active Compounds,” Phosphorus, Sulfur, and Silicon and the Related Elements 193 (2018): 409–414.

[4] V. Zvarych , M. Stasevych , V. Novikov , and M. Vovk , “Synthesis and Study of Antimicrobial Activity of 2‐Dithiocarbamate‐N‐(9,10‐Dioxo‐9,10‐Dihydroanthracenyl)acetamides,” Biointerface Research in Applied Chemistry 11 (2020): 7725–7734.

[5] Q. Zhang , J. Liu , R. Li , et al., “A Network Pharmacology Approach to Investigate the Anticancer Mechanism and Potential Active Ingredients of, *Rheum palmatum*, L. Against Lung Cancer via Induction of Apoptosis,” Frontiers in Pharmacology 11 (2020): 528308, 10.3389/fphar.2020.528308. PMC7672213 33250766

[6] M. Stasevych , V. Zvarych , M. Barus , and M. Bratenko , “2‐Amino‐ and Diamino‐9,10‐Anthracenedione Derivatives: Biofocus and Applied Advantages – A Mini‐Review,” Biointerface Research in Applied Chemistry 11 (2021): 14103–14114, 10.33263/BRIAC.

[7] M. S. Malik , R. I. Alsantali , R. S. Jassas , et al., “Journey of Anthraquinones as Anticancer Agents – a Systematic Review of Recent Literature,” RSC Advances 11 (2021): 35806–35827, 10.1039/D1RA05686G. PMC9043427 35492773

[8] L. Zhao and L. Zheng , “A Review on Bioactive Anthraquinone and Derivatives as the Regulators for ROS,” Molecules 28 (2023): 8139, 10.3390/molecules28248139. PMC10745977 38138627

[9] V. I. Shupeniuk , A. Nepolrai , T. N. Taras , O. P. Sabadakh , M. P. Matkivskyi , and E. R. Luchkevich , “In‐Silico Study of Anthraquinone Derivatives as Possible COVID‐19 Inhibitors,” Journal of Chemistry and Technologies 30 (2022): 151–158.

[10] V. Shupeniuk , “Synthesis and Antimicrobial Activity of Nitrogen‐Containing Anthraquinone Derivatives,” Iraqi Journal of Pharmaceutical Sciences 31 (2022): 193–201, 10.31351/vol31iss2pp193-201.

[11] S. Feng , P. Yin , C. He , S. Pang , and J. N. Shreeve , “Tunable Dimroth Rearrangement of Versatile 1,2,3‐Triazoles Towards High‐Performance Energetic Materials,” Journal of Materials Chemistry A 9 (2021): 12291–12298, 10.1039/D1TA00109D.

[12] M. Jochem , D. Limbach , S. Glang , T. Haspel , and H. Detert , “Experimental and Theoretical Investigation on the Thermal Isomerization Reaction of Tristriazolotriazines,” Journal of Physical Organic Chemistry 35 (2022): e4346, 10.1002/poc.v35.7.

[13] H. S. Mohamed , Z. S. Hamza , A. M. Nagdy , and H. R. A. El‐Mageed , “Computational Studies and DFT Calculations of Synthesized Triazolo Pyrimidine Derivatives: A Review,” Journal of Chemical Reviews 4 (2022): 156–190.

[14] S. Ferrini , J. Z. Chandanshive , S. Lena , et al., “Ruthenium‐Catalyzed Synthesis of 5‐Amino‐1,2,3‐Triazole‐4‐Carboxylates for Triazole‐Based Scaffolds: Beyond the Dimroth Rearrangement,” Journal of Organic Chemistry 80 (2015): 2562–2572, 10.1021/jo502577e. 25654488

[15] C. Wentrup , M. S. Mirzaei , D. Kvaskoff , and A. A. Taherpour , “When a Dimroth Rearrangement Is Not a Dimroth Rearrangement,” Journal of Organic Chemistry 86 (2021): 8286–8294, 10.1021/acs.joc.1c00730. 34077230

[16] M. J. Frisch , G. W. Trucks , H. B. Schlegel , et al., Gaussian 09, Revision A.02 (2016).

[17] S. A. M. Salih , H. A. Basheer , J. V. de Julián‐Ortiz , and H. A. Mohammad‐Salim , “Unveiling the Stereoselectivity and Regioselectivity of the [3+2] Cycloaddition Reaction Between N‐Methyl‐C‐4‐Methylphenyl‐Nitrone and 2‐Propynamide From a MEDT Perspective,” International Journal of Molecular Sciences 24 (2023): 9102, 10.3390/ijms24109102. PMC10218864 37240445

[18] R. J. Hassan and H. H. Abdallah , “Theoretical Study of Diels–Alder Reaction of but‐3‐en‐2‐One With Hexa‐1,2,4‐Triene: A Density Functional Theory Study,” ARO – the Scientific Journal of Koya University 8 (2020): 74–78, 10.14500/aro.10632.

[19] N. Rishu , D. K. Gopalakrishnan , J. Vaitla , and T. Karmakar , “Insights Into the Temperature‐Driven Competitive Pericyclic Transformations of Allyloxy Furan,” Organic & Biomolecular Chemistry 23 (2025): 7110–7114, 10.1039/D5OB01036E. 40658127

