# Supplementary material for: Quantum Chemical Investigation of the Dimroth Rearrangement of 1‐(5‐Imino‐4,5‐dihydro‐1H‐1,2,3‐triazol‐1‐yl)anthracene‐9,10‐dione
Source: ChemistryOpen. 2026 Oct 28;15(11):e70330. doi: 10.1002/open.70330 (PMC13716691; doi:10.1002/open.70330)
Supplement: Supplementary file 1 [file OPEN-15-e70330-s001.pdf]

Supporting Information for

Quantum Chemical Investigation of the Dimroth Rearrangement of 1-(5-Imino-4,5-dihydro-1H-1,2,3-triazol-1-yl)anthracene-9,10-dione

Dmytro Melnyk*[a], Vasyl Shupeniuk[b], Tetyana Taras[b], Volodymyr Kotsyubynsky[b]

Energies and Cartesian coordinates of all the stationary points

1. Energies of all the stationary points

	E (a.u.)	G (a.u.)	Imaginary frequency (cm^{-1})
**1**	-1140.4963	-1140.2433	–
**2**	-1140.5055	-1140.2494	–
**TS2_3**	-1140.4593	-1140.2016	-1357.08
**3**	-1140.5300	-1140.2733	–
**T3_4**	-1140.4991	-1140.2457	-222.20
**4**	-1140.5196	-1140.2676	–
**T4_5**	-1140.5053	-1140.2532	-117.98
**5**	-1140.5164	-1140.2651	–
**6**	-1140.5153	-1140.2622	–
**6'**	-1140.477	-1140.2193	–
**7**	-1140.5452	-1140.2860	–

2. Cartesian coordinates of all the stationary points

1

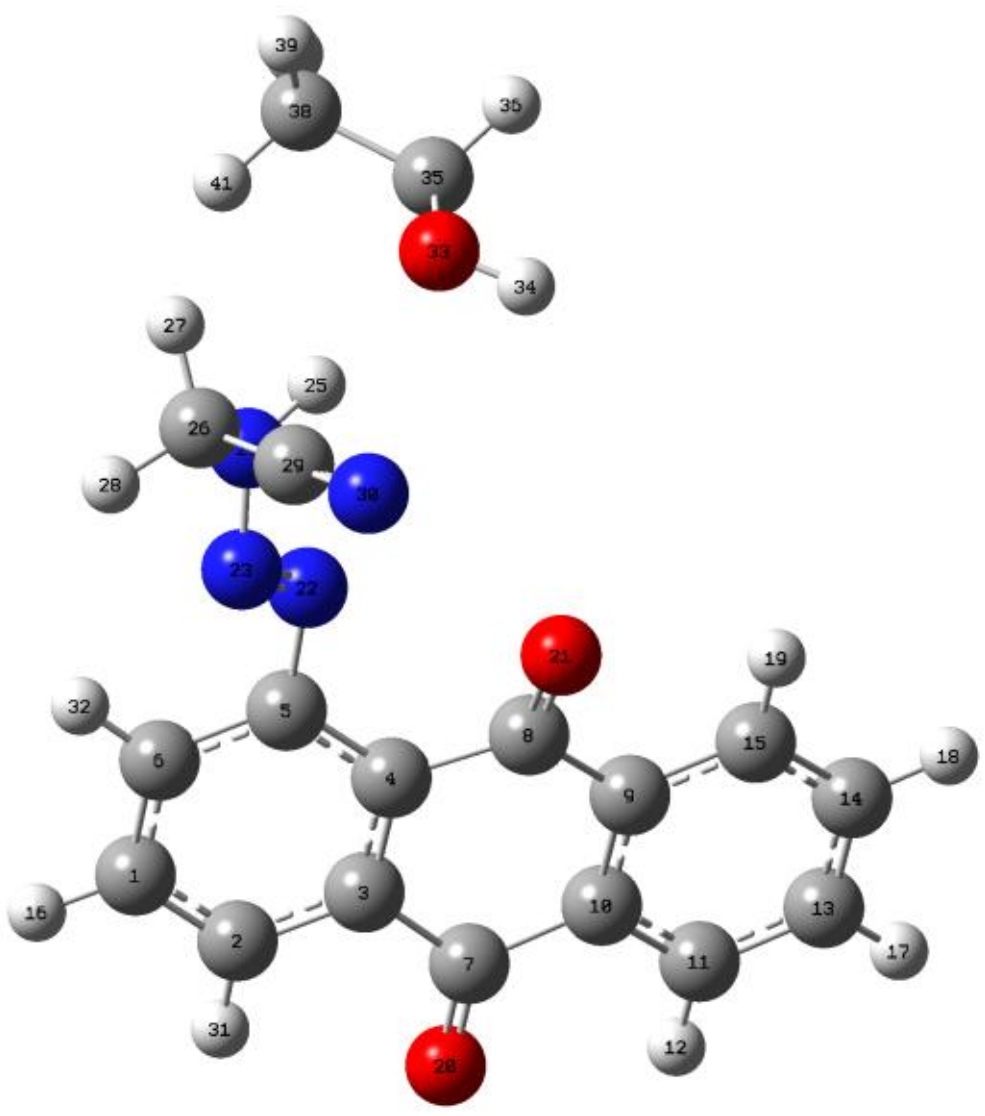

Center Number	Atomic Number	Atomic Type	Coordinates (Angstroms) X	Y	Z
1	6	0	-0.893466	3.534410	-1.027941
2	6	0	-2.136445	2.944418	-0.823637
3	6	0	-2.218585	1.620388	-0.392115
4	6	0	-1.048136	0.871304	-0.133965
5	6	0	0.210361	1.471901	-0.373877
6	6	0	0.266842	2.799857	-0.822974
7	6	0	-3.579031	1.021255	-0.239141
8	6	0	-1.162873	-0.495101	0.445995
9	6	0	-2.505951	-1.149686	0.414317
10	6	0	-3.665139	-0.422305	0.097412
11	6	0	-4.911919	-1.056799	0.112688
12	1	0	-5.795997	-0.478910	-0.125004
13	6	0	-5.004409	-2.407758	0.426458
14	6	0	-3.851058	-3.132896	0.739491
15	6	0	-2.610103	-2.504963	0.742031
16	1	0	-0.827673	4.560569	-1.370021
17	1	0	-5.970906	-2.897744	0.429786
18	1	0	-3.923187	-4.186163	0.984143
19	1	0	-1.711381	-3.053536	0.993225
20	8	0	-4.587287	1.694071	-0.413388
21	8	0	-0.213221	-1.072012	0.957957
22	7	0	1.391505	0.705426	-0.287329
23	7	0	2.369665	1.326882	0.199472
24	7	0	3.524070	0.668295	0.124941
25	1	0	3.519116	-0.327017	-0.143738
26	6	0	4.616361	1.182409	0.920848
27	1	0	5.558740	0.827885	0.499931
28	1	0	4.618611	2.272889	0.874005
29	6	0	4.556605	0.774281	2.338830

30	7	0	4.500662	0.446554	3.442878
31	1	0	-3.051465	3.492391	-1.005297
32	1	0	1.236282	3.234692	-1.030561
33	8	0	3.826486	-2.179015	-0.470018
34	1	0	3.270961	-2.691224	0.129024
35	6	0	3.722455	-2.746743	-1.794717
36	1	0	3.956907	-3.815018	-1.747575
37	1	0	2.695567	-2.636755	-2.159736
38	6	0	4.696420	-2.026278	-2.706088
39	1	0	5.722550	-2.142228	-2.348158
40	1	0	4.636245	-2.440866	-3.715588
41	1	0	4.464929	-0.959564	-2.762279
1	6	0	-0.893466	3.534410	-1.027941
2	6	0	-2.136445	2.944418	-0.823637
3	6	0	-2.218585	1.620388	-0.392115
4	6	0	-1.048136	0.871304	-0.133965
5	6	0	0.210361	1.471901	-0.373877
6	6	0	0.266842	2.799857	-0.822974
7	6	0	-3.579031	1.021255	-0.239141
8	6	0	-1.162873	-0.495101	0.445995

2

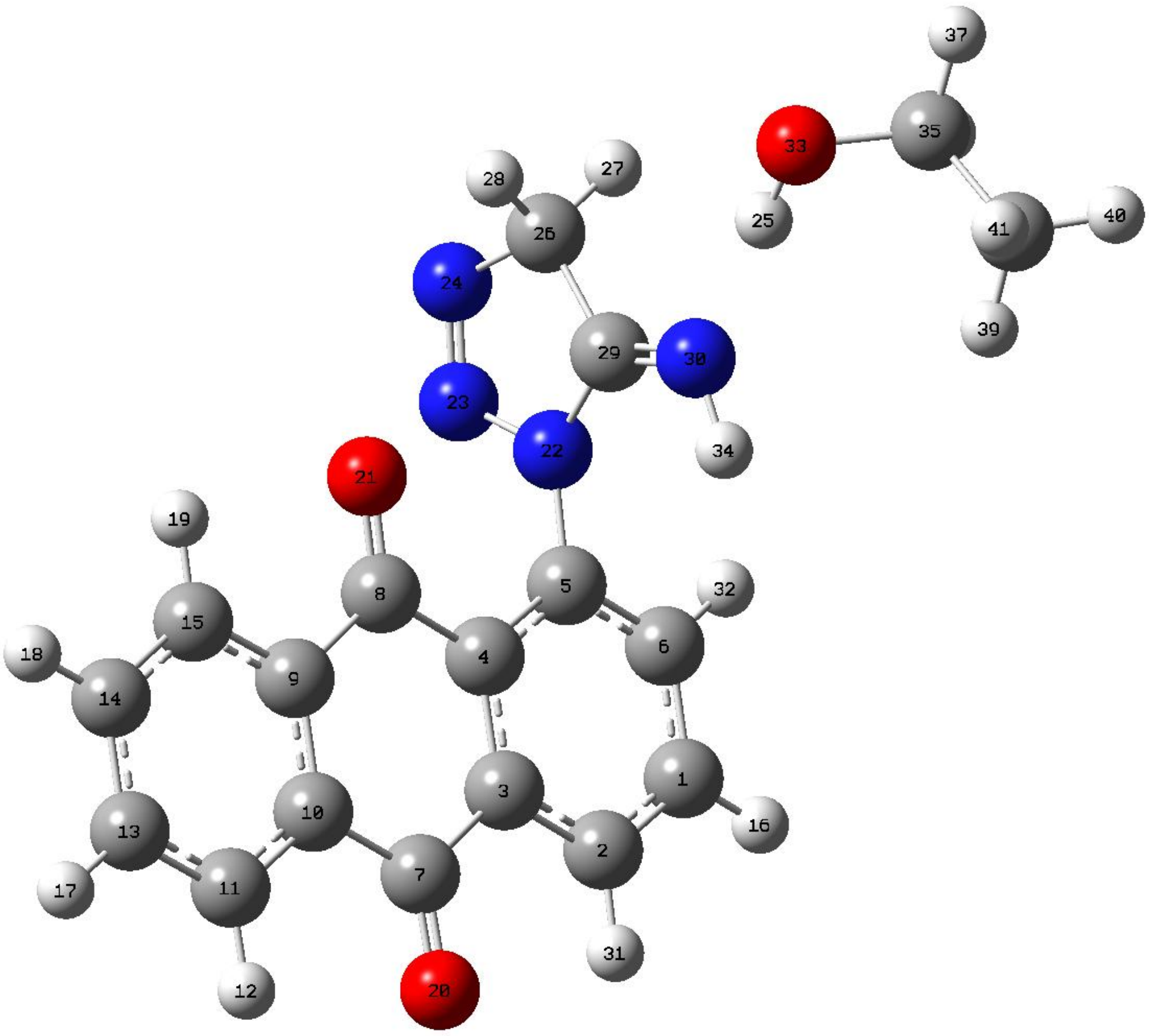

Center Number	Atomic Number	Atomic Type	Coordinates (Angstroms) X	Y	Z
1	6	0	-0.336773	3.547002	-0.109537
2	6	0	-1.528794	3.000609	-0.56174
3	6	0	-1.810668	1.645871	-0.364009
4	6	0	-0.88678	0.807197	0.29213
5	6	0	0.312757	1.385974	0.757833
6	6	0	0.580561	2.737385	0.555795
7	6	0	-3.123577	1.130574	-0.865008
8	6	0	-1.185764	-0.653979	0.454647
9	6	0	-2.518032	-1.145695	0.001951
10	6	0	-3.443894	-0.297994	-0.630252
11	6	0	-4.678698	-0.804117	-1.048606
12	1	0	-5.378705	-0.138095	-1.536584
13	6	0	-4.993762	-2.141379	-0.837003
14	6	0	-4.074336	-2.985201	-0.20779
15	6	0	-2.842739	-2.49079	0.206775
16	1	0	-0.121493	4.597223	-0.262307
17	1	0	-5.952508	-2.528848	-1.160613
18	1	0	-4.320145	-4.027496	-0.042644
19	1	0	-2.12163	-3.135074	0.692662
20	8	0	-3.904287	1.874242	-1.442208
21	8	0	-0.371988	-1.431147	0.93078
22	7	0	1.295429	0.628263	1.458612
23	7	0	1.132098	0.34869	2.815336

24	7	0	2.012576	-0.427564	3.225518
25	1	0	4.224963	-1.152729	-0.915184
26	6	0	2.917	-0.800592	2.126019
27	1	0	3.936083	-0.490505	2.370789
28	1	0	2.915656	-1.885773	2.00283
29	6	0	2.363371	-0.077972	0.925793
30	7	0	2.796392	-0.121173	-0.272655
31	1	0	-2.262062	3.610908	-1.072479
32	1	0	1.510667	3.146138	0.931234
33	8	0	4.973866	-1.714721	-1.212162
34	1	0	2.248541	0.454733	-0.911412
35	6	0	6.024857	-0.868178	-1.677798
36	1	0	6.238617	-0.085831	-0.937158
37	1	0	6.91721	-1.496242	-1.750401
38	6	0	5.722554	-0.234507	-3.032403
39	1	0	4.840869	0.41061	-2.975755
40	1	0	6.567702	0.375959	-3.366694
41	1	0	5.536337	-1.004724	-3.786039

T2_3

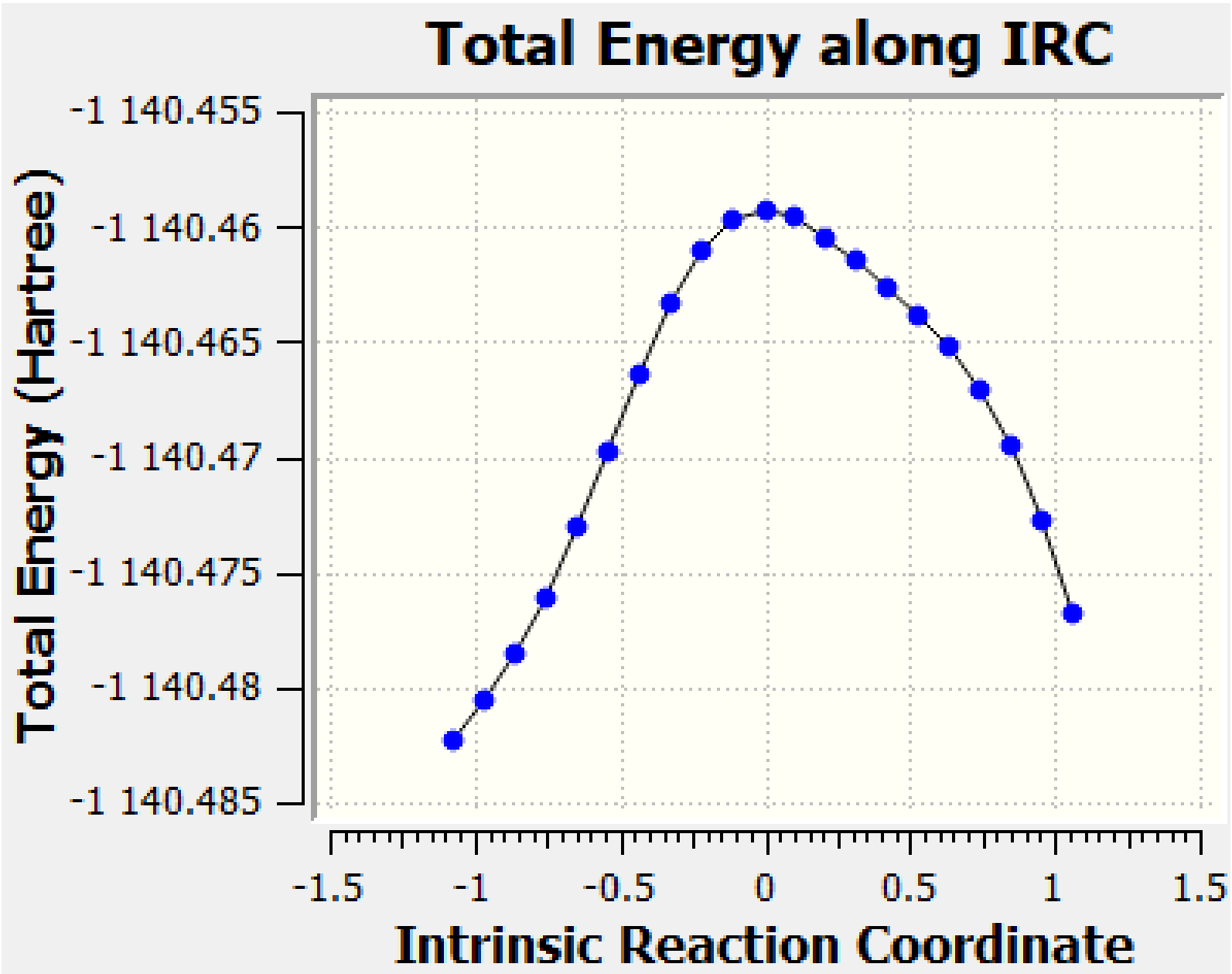

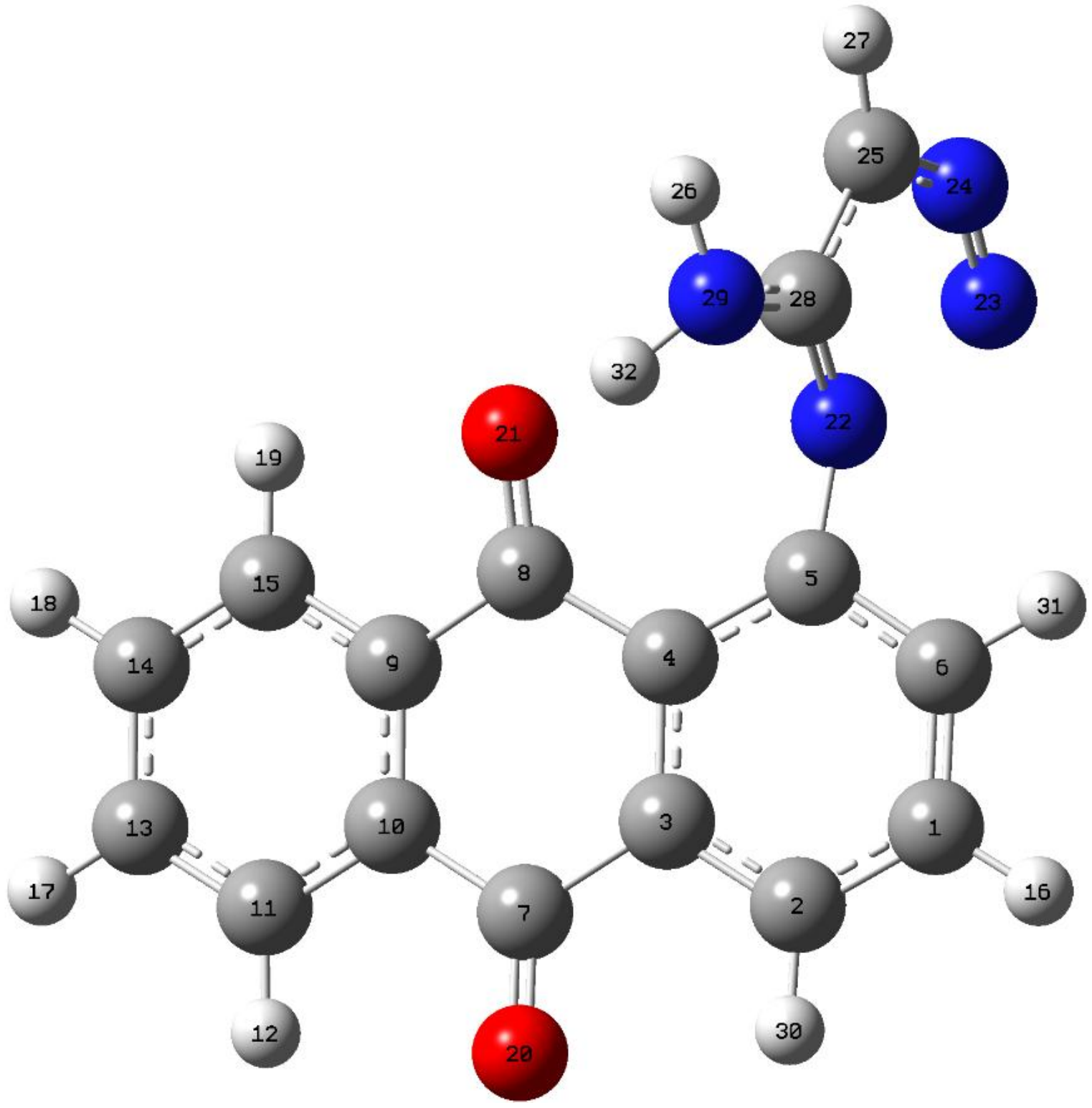

Center Number	Atomic Number	Atomic Type	Coordinates (Angstroms) X	Y	Z
1	6	0	-0.676518	3.70085	-0.729951
2	6	0	-1.835057	2.947943	-0.865378
3	6	0	-1.85543	1.606403	-0.476895
4	6	0	-0.695043	0.988762	0.03594
5	6	0	0.456772	1.782312	0.214731
6	6	0	0.45985	3.12245	-0.171738
7	6	0	-3.146213	0.861386	-0.606474
8	6	0	-0.697357	-0.486971	0.289623
9	6	0	-2.01779	-1.178106	0.322525
10	6	0	-3.190793	-0.536677	-0.110554
11	6	0	-4.407932	-1.225278	-0.091491
12	1	0	-5.300974	-0.719875	-0.436339

13	6	0	-4.461173	-2.536888	0.366353
14	6	0	-3.295178	-3.174862	0.798755
15	6	0	-2.078546	-2.501851	0.769128
16	1	0	-0.661559	4.741637	-1.029176
17	1	0	-5.407392	-3.064454	0.386837
18	1	0	-3.337619	-4.196836	1.156445
19	1	0	-1.167847	-2.987434	1.095293
20	8	0	-4.13437	1.400616	-1.085484
21	8	0	0.336484	-1.122675	0.429715
22	7	0	1.634008	1.273833	0.818272
23	7	0	1.632308	0.873212	2.166815
24	7	0	2.773514	0.400735	2.441207
25	6	0	3.597502	0.387538	1.311895
26	1	0	4.654989	0.570706	1.475268
27	1	0	3.873971	-0.831114	0.505579
28	6	0	2.848692	1.004662	0.231483
29	7	0	3.215472	1.050958	-1.026916
30	1	0	-2.740372	3.38559	-1.265199
31	1	0	1.355915	3.709841	-0.012387
32	1	0	2.484065	1.394135	-1.644054
33	8	0	4.189602	-1.31159	-0.527094
34	1	0	3.905861	-0.469417	-1.058743
35	6	0	3.385276	-2.47693	-0.908407
36	1	0	2.330713	-2.205866	-0.831923
37	1	0	3.610917	-3.237517	-0.161667
38	6	0	3.763487	-2.927962	-2.303633
39	1	0	4.822088	-3.190219	-2.357976
40	1	0	3.175718	-3.811056	-2.567444
41	1	0	3.553408	-2.150775	-3.042739

3

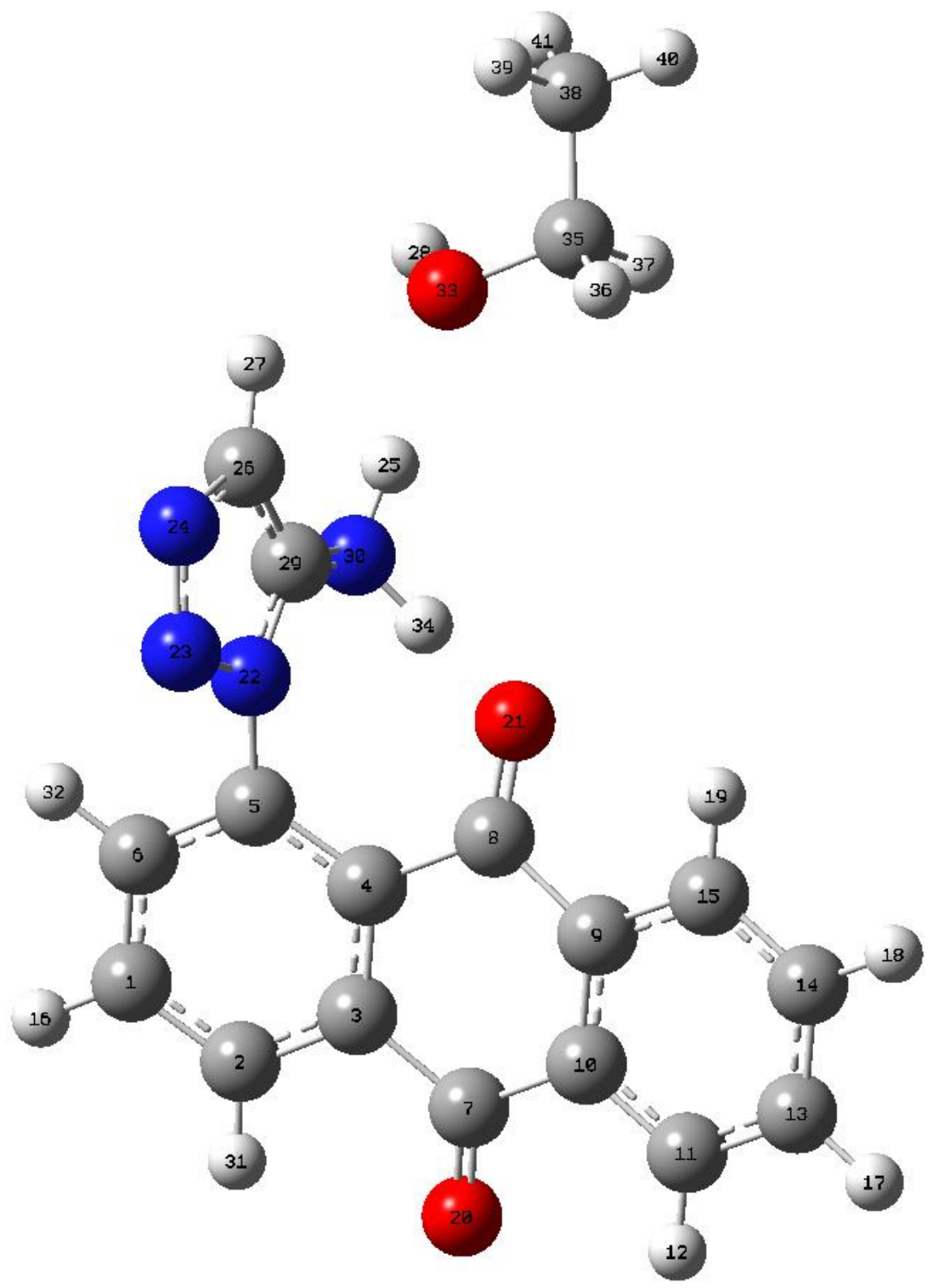

Center Number	Atomic Number	Atomic Type	Coordinates (Angstroms) X	Y	Z
1	6	0	1.514882	-3.52774	-0.911367
2	6	0	2.507379	-2.574755	-1.094475
3	6	0	2.313986	-1.262061	-0.660671
4	6	0	1.100119	-0.873641	-0.053543
5	6	0	0.105873	-1.856474	0.127443
6	6	0	0.322126	-3.168587	-0.292037
7	6	0	3.449184	-0.301098	-0.825157
8	6	0	0.909739	0.548253	0.368709
9	6	0	2.108722	1.430839	0.397615
10	6	0	3.321515	1.034027	-0.190682
11	6	0	4.414635	1.906115	-0.186962
12	1	0	5.339354	1.588975	-0.651934
13	6	0	4.307534	3.156694	0.410989
14	6	0	3.102411	3.550524	0.999488
15	6	0	2.005646	2.695766	0.984899
16	1	0	1.666761	-4.548082	-1.241033
17	1	0	5.159345	3.826284	0.419802
18	1	0	3.020457	4.524986	1.466168
19	1	0	1.064059	2.99348	1.427986
20	8	0	4.458092	-0.624337	-1.436281
21	8	0	-0.19217	0.988739	0.662559
22	7	0	-1.129937	-1.609815	0.799807
23	7	0	-1.328147	-2.139657	2.060137

24	7	0	-2.565687	-1.908169	2.371587
25	1	0	-3.298214	-0.02181	-1.069903
26	6	0	-3.19017	-1.249646	1.362308
27	1	0	-4.225655	-0.956732	1.412321
28	1	0	-5.559697	0.548948	-2.077246
29	6	0	-2.279763	-1.049093	0.333837
30	7	0	-2.416339	-0.514485	-0.921563
31	1	0	3.448004	-2.832583	-1.563235
32	1	0	-0.457533	-3.901363	-0.12697
33	8	0	-5.014288	0.839224	-1.336885
34	1	0	-1.623345	0.030362	-1.235275
35	6	0	-5.263209	2.238004	-1.095346
36	1	0	-4.570837	2.516703	-0.299085
37	1	0	-5.000225	2.817814	-1.98694
38	6	0	-6.700741	2.509025	-0.680281
39	1	0	-6.957396	1.949907	0.222931
40	1	0	-6.837588	3.575182	-0.476977
41	1	0	-7.399021	2.226897	-1.474122

T3_4

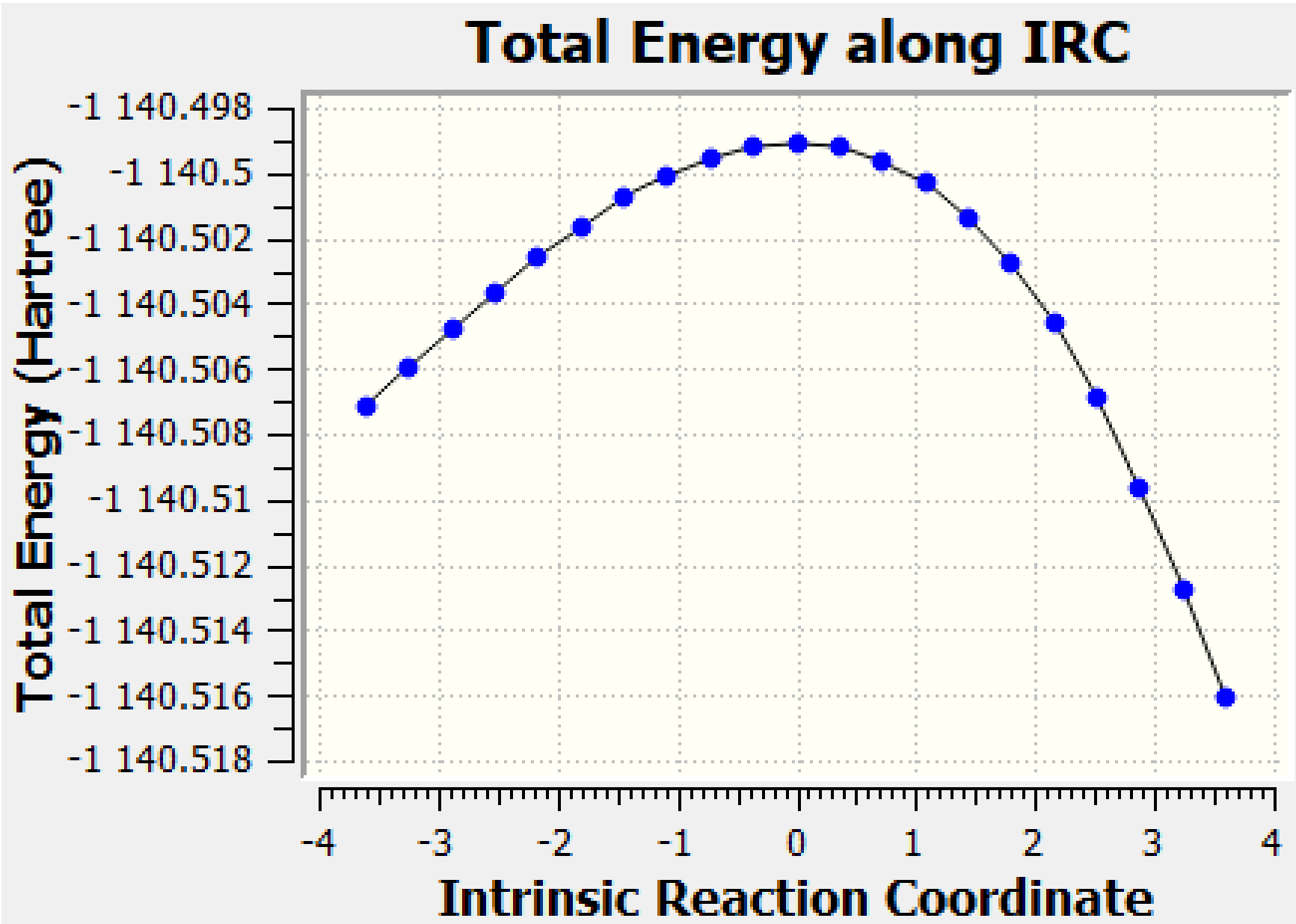

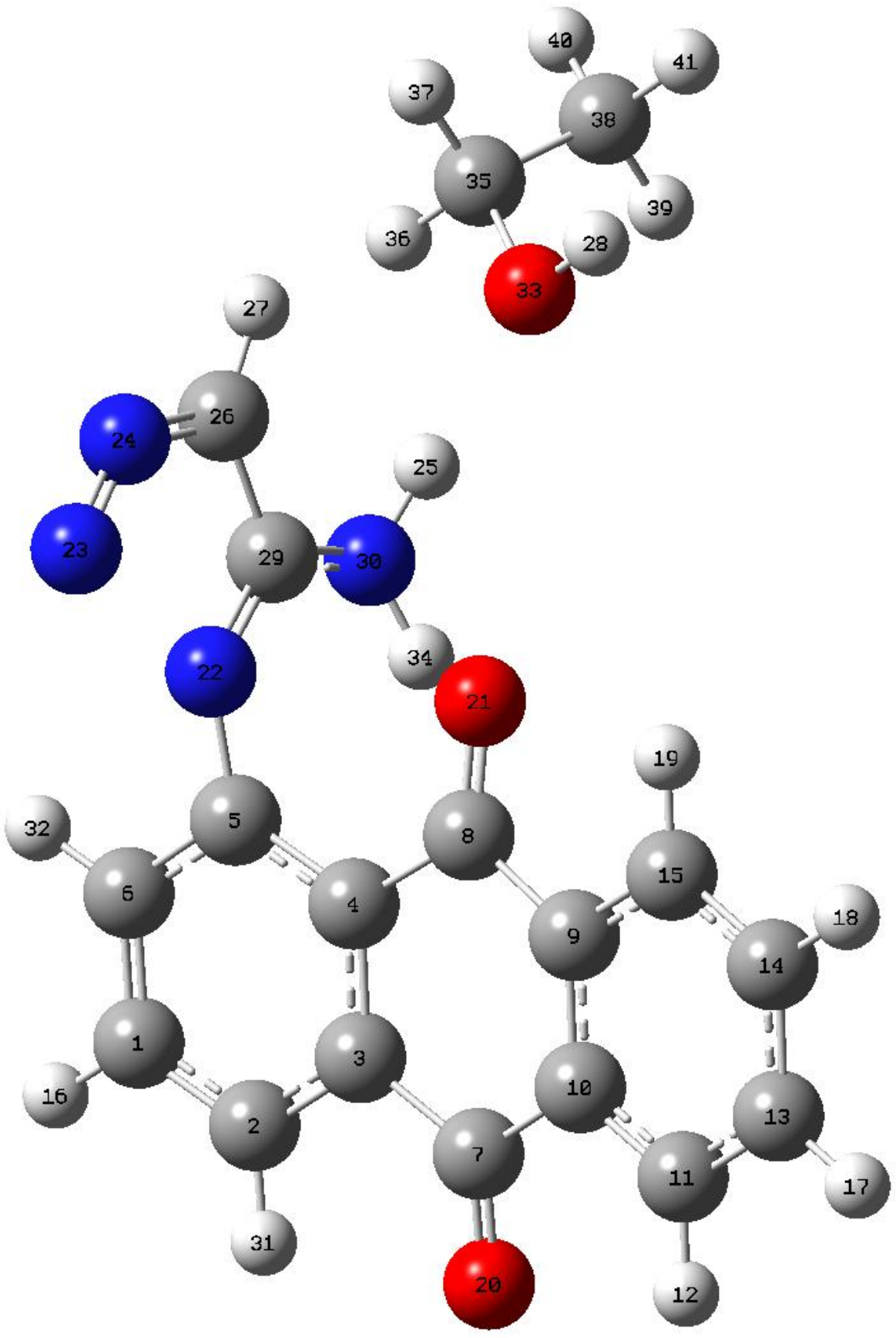

Center Number	Atomic Number	Atomic Type	Coordinates (Angstroms) X	Y	Z
1	6	0	-1.12259	3.463965	-1.187881
2	6	0	-2.193048	2.59944	-1.398578
3	6	0	-2.179006	1.322585	-0.841744
4	6	0	-1.074192	0.871254	-0.07696
5	6	0	0.019134	1.758228	0.130851
6	6	0	-0.042169	3.05145	-0.42453
7	6	0	-3.383218	0.466287	-1.06661
8	6	0	-1.092906	-0.501237	0.499613
9	6	0	-2.369828	-1.277127	0.419701

10	6	0	-3.460538	-0.824174	-0.3395
11	6	0	-4.626066	-1.594668	-0.417078
12	1	0	-5.454281	-1.231497	-1.012166
13	6	0	-4.711675	-2.802206	0.26533
14	6	0	-3.627428	-3.253581	1.023703
15	6	0	-2.461613	-2.498952	1.094294
16	1	0	-1.135132	4.461043	-1.612268
17	1	0	-5.618196	-3.39324	0.209105
18	1	0	-3.693679	-4.19494	1.556581
19	1	0	-1.612417	-2.842474	1.670773
20	8	0	-4.286607	0.830556	-1.809684
21	8	0	-0.112031	-1.009011	1.027615
22	7	0	1.111732	1.471591	0.933742
23	7	0	1.677413	1.878076	2.953952
24	7	0	2.673854	1.286292	2.774096
25	1	0	3.232614	-0.404876	-0.668027
26	6	0	3.140797	0.661097	1.705777
27	1	0	4.090839	0.159285	1.688504
28	1	0	4.678107	-2.475498	-0.371761
29	6	0	2.17938	0.791619	0.628927
30	7	0	2.469598	0.266138	-0.590109
31	1	0	-3.051463	2.904686	-1.98198
32	1	0	0.788318	3.721018	-0.236169
33	8	0	4.693356	-1.631014	-0.836947
34	1	0	1.718094	0.170099	-1.255935
35	6	0	6.061256	-1.27143	-1.11441
36	1	0	6.008043	-0.27828	-1.563766
37	1	0	6.616219	-1.184825	-0.173935
38	6	0	6.74157	-2.25387	-2.05471
39	1	0	6.201768	-2.323416	-3.002394
40	1	0	7.764156	-1.925792	-2.263152
41	1	0	6.79394	-3.252301	-1.610221

4

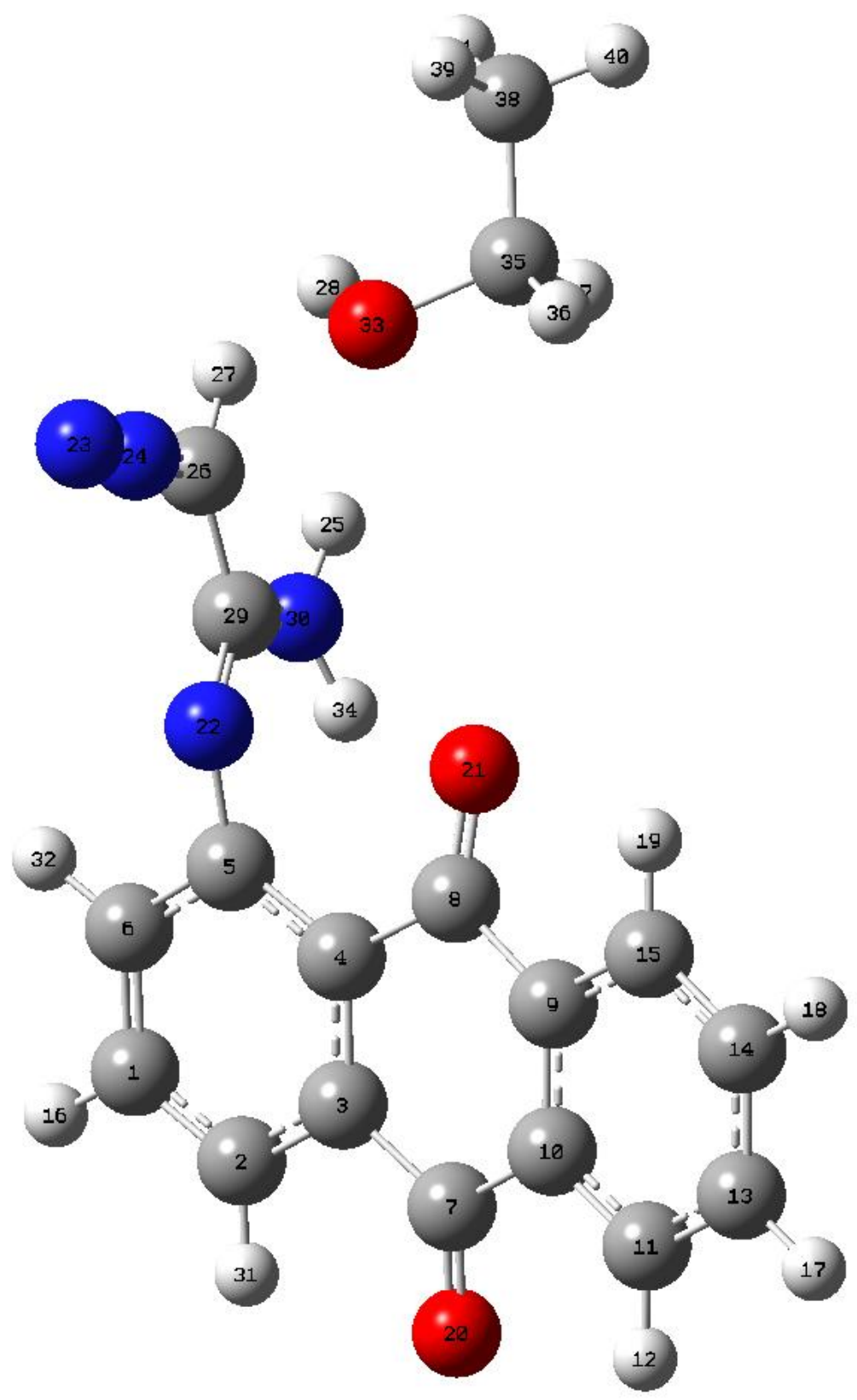

Center Number	Atomic Number	Atomic Type	Coordinates (Angstroms) X	Y	Z
1	6	0	1.063311	-3.582121	-0.493691
2	6	0	2.22401	-2.857259	-0.75815
3	6	0	2.273943	-1.497735	-0.464244
4	6	0	1.149509	-0.827915	0.085199
5	6	0	-0.023239	-1.576182	0.382165
6	6	0	-0.032339	-2.954657	0.075271
7	6	0	3.557971	-0.784645	-0.733608
8	6	0	1.210151	0.636955	0.31641
9	6	0	2.543961	1.308792	0.199321
10	6	0	3.664245	0.634502	-0.31194
11	6	0	4.886945	1.304384	-0.435047
12	1	0	5.737477	0.769577	-0.838418
13	6	0	4.999131	2.632277	-0.04116
14	6	0	3.885169	3.304382	0.47069
15	6	0	2.664109	2.648448	0.58288
16	1	0	1.021026	-4.641803	-0.718433
17	1	0	5.949012	3.146015	-0.131598
18	1	0	3.971744	4.339854	0.77886
19	1	0	1.792458	3.160937	0.969197
20	8	0	4.501666	-1.357387	-1.267026
21	8	0	0.221398	1.309624	0.583696
22	7	0	-1.102899	-1.049145	1.075026
23	7	0	-3.07957	-0.162979	3.738271

24	7	0	-3.220872	-0.247084	2.617901
25	1	0	-3.395464	-0.518881	-1.17298
26	6	0	-3.367118	-0.349192	1.329898
27	1	0	-4.329176	-0.077354	0.919686
28	1	0	-5.855809	-0.315861	-1.802054
29	6	0	-2.240643	-0.796885	0.516457
30	7	0	-2.528515	-0.912913	-0.814167
31	1	0	3.097036	-3.334465	-1.182874
32	1	0	-0.923858	-3.522351	0.315719
33	8	0	-5.166078	0.251023	-1.437026
34	1	0	-1.766514	-1.027912	-1.464135
35	6	0	-5.351507	1.591849	-1.935543
36	1	0	-4.513323	2.162656	-1.532385
37	1	0	-5.263842	1.591735	-3.027356
38	6	0	-6.676931	2.195719	-1.499676
39	1	0	-6.756637	2.216672	-0.409965
40	1	0	-6.760874	3.221059	-1.871437
41	1	0	-7.520222	1.624584	-1.899588

T4_5

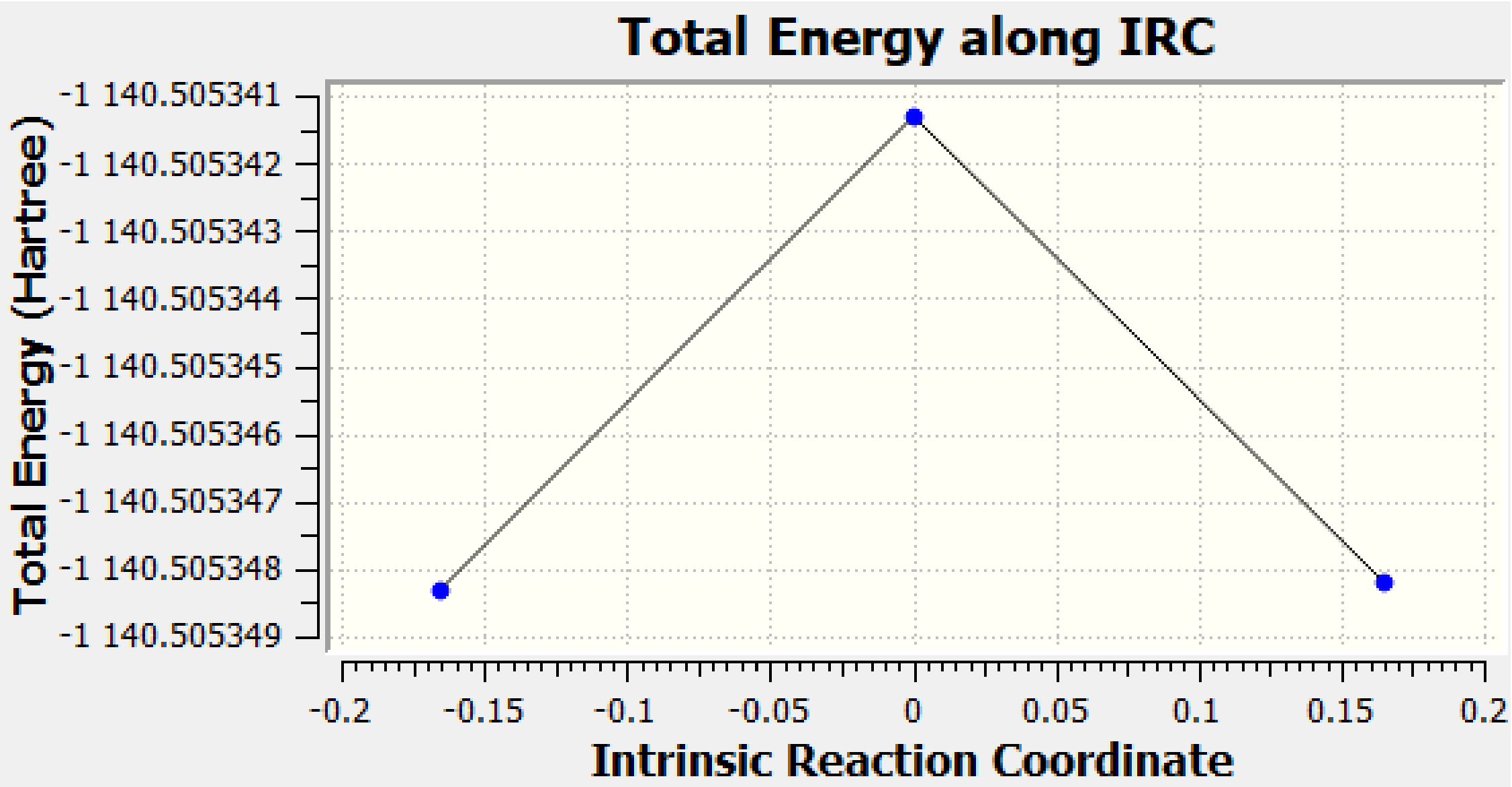

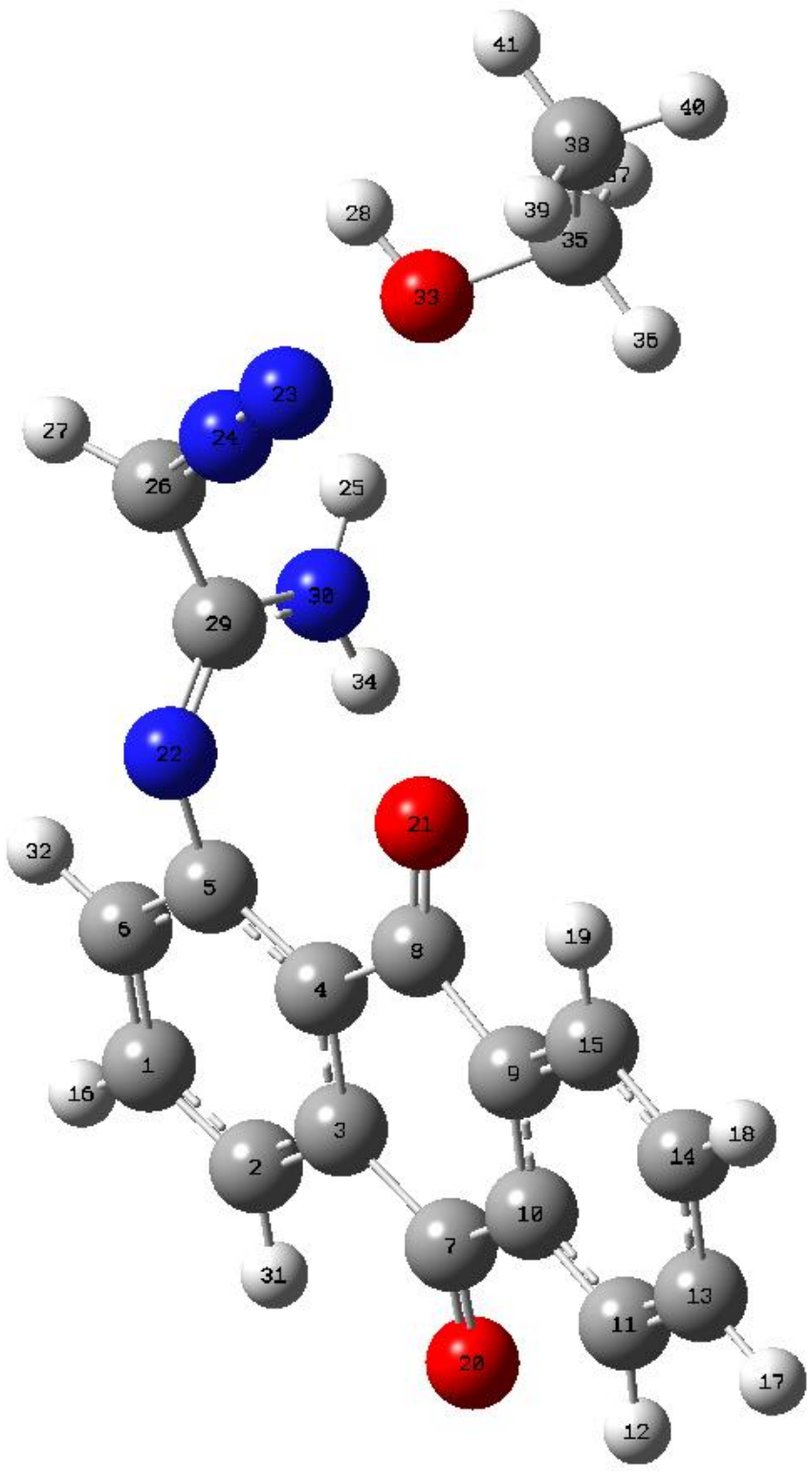

Center Number	Atomic Number	Atomic Type	Coordinates (Angstroms) X	Y	Z
1	6	0	1.168551	3.706301	0.014345
2	6	0	2.263994	2.955165	0.434734
3	6	0	2.262984	1.573254	0.2673
4	6	0	1.149629	0.908803	-0.308848
5	6	0	0.04404	1.681956	-0.753715
6	6	0	0.084949	3.080348	-0.580403
7	6	0	3.482585	0.827671	0.70091
8	6	0	1.156647	-0.571493	-0.426933
9	6	0	2.437857	-1.290389	-0.139577

10	6	0	3.546778	-0.626065	0.408718
11	6	0	4.718261	-1.338114	0.690246
12	1	0	5.560754	-0.810184	1.11866
13	6	0	4.791433	-2.698969	0.418345
14	6	0	3.688855	-3.361301	-0.12948
15	6	0	2.517601	-2.662209	-0.39997
16	1	0	1.166711	4.782986	0.139401
17	1	0	5.702341	-3.245876	0.631624
18	1	0	3.745235	-4.422487	-0.342295
19	1	0	1.654502	-3.165439	-0.816356
20	8	0	4.408866	1.401497	1.262092
21	8	0	0.161989	-1.214342	-0.740434
22	7	0	-1.027305	1.151563	-1.472233
23	7	0	-3.542057	-1.959436	-2.266033
24	7	0	-3.420771	-0.846959	-2.053214
25	1	0	-3.386518	0.739447	0.743994
26	6	0	-3.278078	0.411555	-1.802491
27	1	0	-3.944637	1.095384	-2.307871
28	1	0	-5.883584	0.693135	1.242184
29	6	0	-2.155236	0.871094	-0.923462
30	7	0	-2.453708	0.952662	0.395638
31	1	0	3.126793	3.428102	0.88434
32	1	0	-0.756179	3.662579	-0.938597
33	8	0	-5.053335	0.392715	1.629907
34	1	0	-1.764182	1.287094	1.052772
35	6	0	-5.305883	-0.788312	2.416889
36	1	0	-4.336128	-1.062873	2.83564
37	1	0	-5.970147	-0.533659	3.249839
38	6	0	-5.883525	-1.929838	1.594912
39	1	0	-5.213073	-2.199852	0.775279
40	1	0	-6.027641	-2.810791	2.227215
41	1	0	-6.856171	-1.658086	1.173424

5

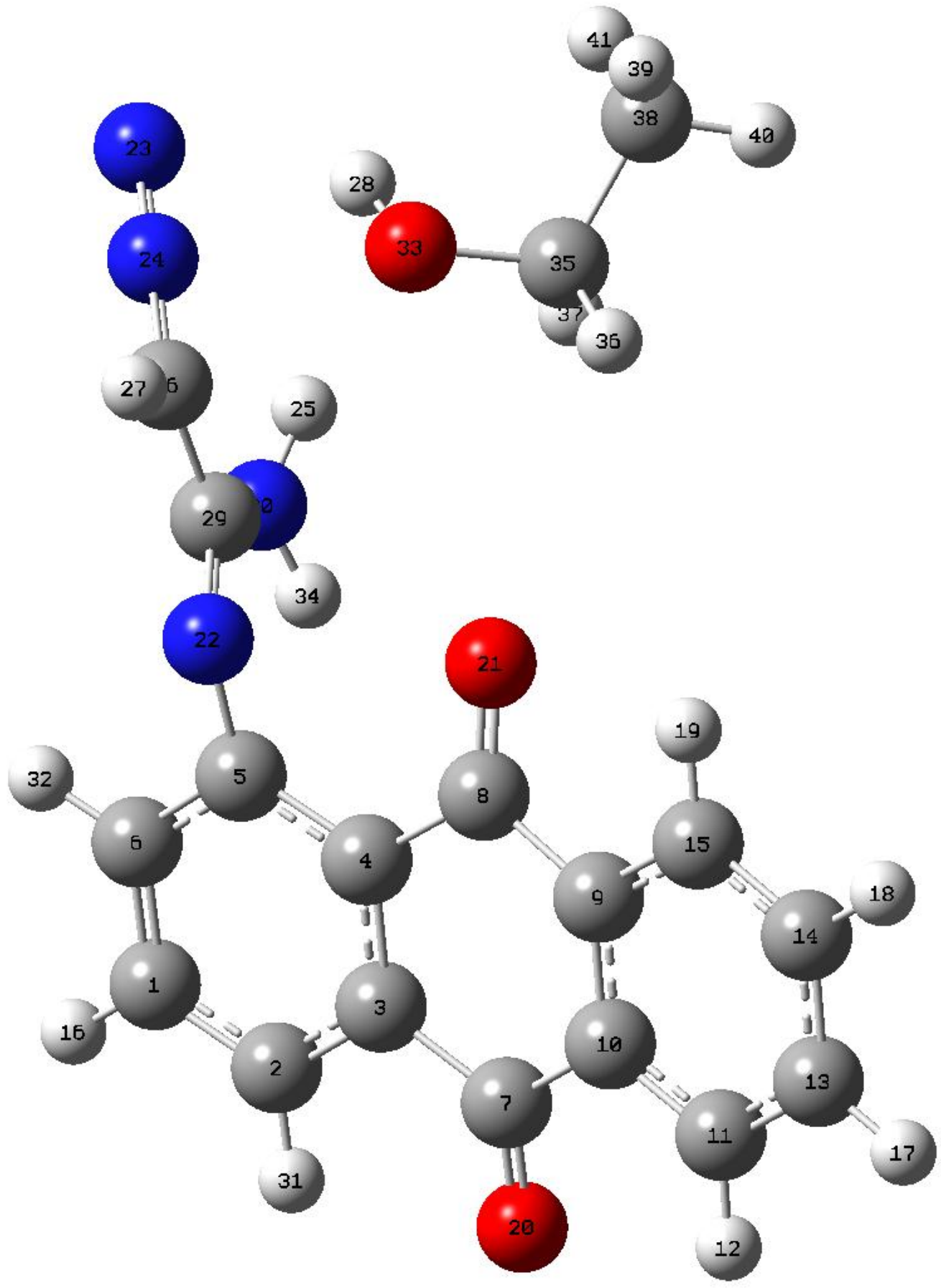

Center Number	Atomic Number	Atomic Type	Coordinates (Angstroms) X	Y	Z
1	6	0	-0.941158	3.485282	-1.011077
2	6	0	-2.076117	2.711118	-1.244371
3	6	0	-2.184064	1.44876	-0.667821
4	6	0	-1.143646	0.925239	0.142253
5	6	0	0.000944	1.728679	0.401629
6	6	0	0.070838	3.006352	-0.195383
7	6	0	-3.440456	0.682429	-0.922502
8	6	0	-1.256537	-0.452718	0.681483
9	6	0	-2.581578	-1.141342	0.566926
10	6	0	-3.623025	-0.605488	-0.207489
11	6	0	-4.837766	-1.29214	-0.315372
12	1	0	-5.626626	-0.866756	-0.922627
13	6	0	-5.02116	-2.497184	0.351991
14	6	0	-3.985946	-3.030822	1.125326
15	6	0	-2.771634	-2.360503	1.225405
16	1	0	-0.854018	4.470308	-1.455216
17	1	0	-5.965552	-3.022635	0.272063
18	1	0	-4.128243	-3.970299	1.64667
19	1	0	-1.95986	-2.768398	1.813843
20	8	0	-4.302488	1.112687	-1.680465
21	8	0	-0.315352	-1.041632	1.199763
22	7	0	0.980086	1.373133	1.317813

23	7	0	5.498042	0.427557	1.70685
24	7	0	4.386859	0.595822	1.861804
25	1	0	3.434043	0.331298	-0.534073
26	6	0	3.115955	0.789747	2.063153
27	1	0	2.79181	0.811521	3.092339
28	1	0	5.623225	-0.594309	-1.508455
29	6	0	2.181484	1.041735	0.972901
30	7	0	2.643557	0.936471	-0.313061
31	1	0	-2.885455	3.076326	-1.862303
32	1	0	0.937846	3.621128	0.017337
33	8	0	4.749247	-0.903819	-1.243368
34	1	0	1.949172	0.985453	-1.044466
35	6	0	4.355152	-1.986126	-2.109778
36	1	0	3.344283	-2.249511	-1.793645
37	1	0	4.301205	-1.628662	-3.144003
38	6	0	5.284378	-3.184749	-2.000982
39	1	0	5.321182	-3.557256	-0.974283
40	1	0	4.931631	-3.992367	-2.649059
41	1	0	6.300309	-2.924223	-2.31315

6

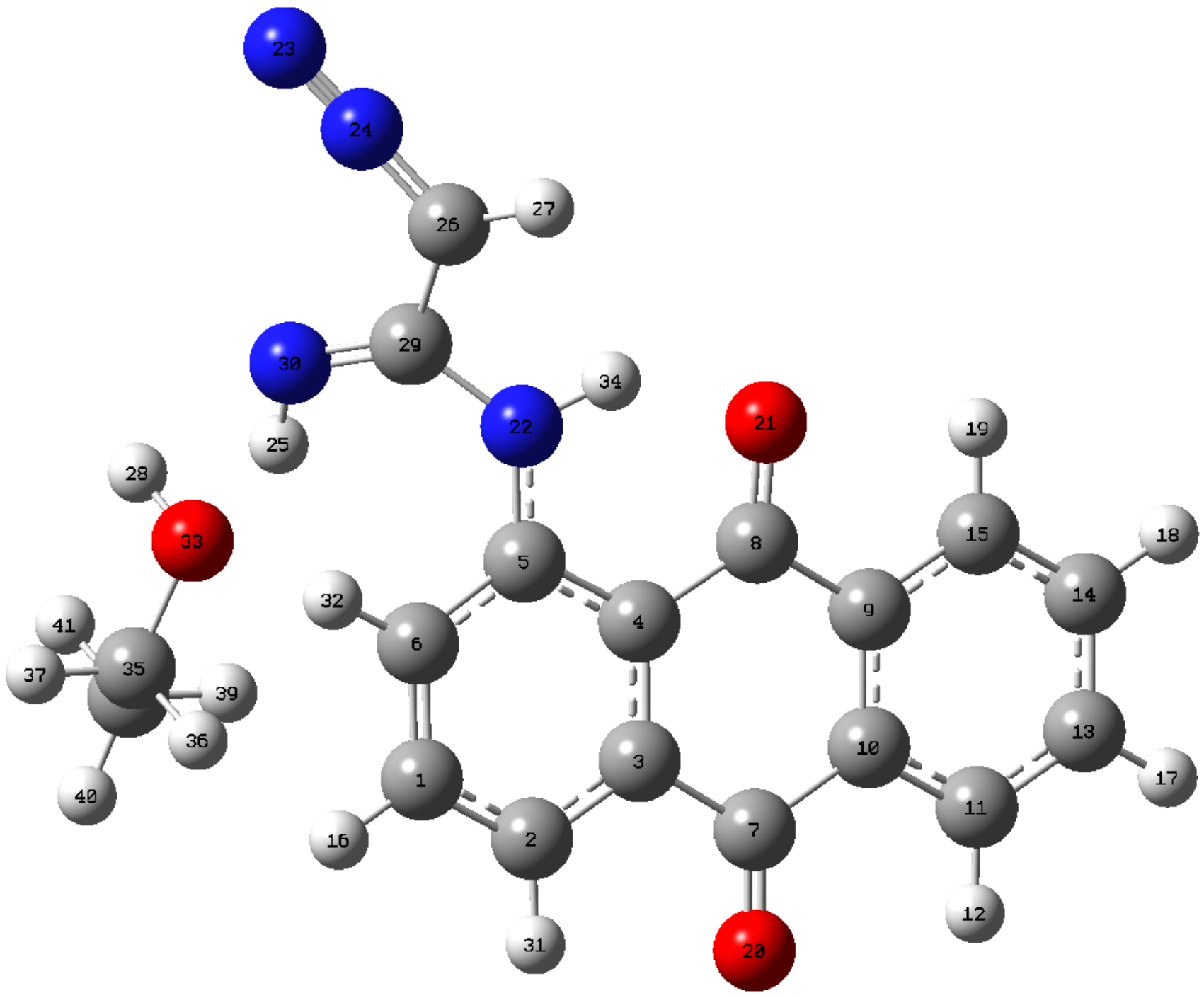

Center Number	Atomic Number	Atomic Type	Coordinates (Angstroms) X	Y	Z
1	6	0	-0.665155	1.887155	-2.022925
2	6	0	0.700108	1.998208	-1.755418
3	6	0	1.348105	0.990214	-1.054754
4	6	0	0.640128	-0.150787	-0.592686
5	6	0	-0.763633	-0.234364	-0.840296
6	6	0	-1.386464	0.797161	-1.574525
7	6	0	2.811098	1.160061	-0.80062
8	6	0	1.365926	-1.227337	0.103826
9	6	0	2.810585	-1.034127	0.411514
10	6	0	3.50693	0.106028	-0.022414
11	6	0	4.866101	0.248096	0.275608
12	1	0	5.386973	1.132107	-0.069421
13	6	0	5.528692	-0.733251	1.003691
14	6	0	4.836993	-1.868167	1.436271
15	6	0	3.487028	-2.019182	1.139137
16	1	0	-1.168637	2.657102	-2.595704
17	1	0	6.581053	-0.618057	1.234875
18	1	0	5.352786	-2.633561	2.004128
19	1	0	2.941969	-2.895007	1.466014
20	8	0	3.41313	2.143802	-1.213561
21	8	0	0.819478	-2.287235	0.43557
22	7	0	-1.47882	-1.339478	-0.421707

23	7	0	-5.565522	-3.433506	0.273689
24	7	0	-4.499871	-3.183006	-0.009293
25	1	0	-3.298968	0.373561	0.149681
26	6	0	-3.277924	-2.865924	-0.333183
27	1	0	-2.645271	-3.675061	-0.665409
28	1	0	-3.851954	2.203639	1.955982
29	6	0	-2.866861	-1.467208	-0.24377
30	7	0	-3.72233	-0.540918	-0.007019
31	1	0	1.268932	2.853227	-2.094499
32	1	0	-2.437462	0.718036	-1.813127
33	8	0	-3.154818	2.274321	1.293567
34	1	0	-0.891537	-2.098243	-0.077774
35	6	0	-2.984326	3.662783	0.961348
36	1	0	-2.22216	3.677947	0.180344
37	1	0	-3.911662	4.058462	0.530471
38	6	0	-2.548119	4.5029	2.152847
39	1	0	-1.610437	4.128039	2.571126
40	1	0	-2.39785	5.542415	1.846286
41	1	0	-3.307585	4.49308	2.940844

6'

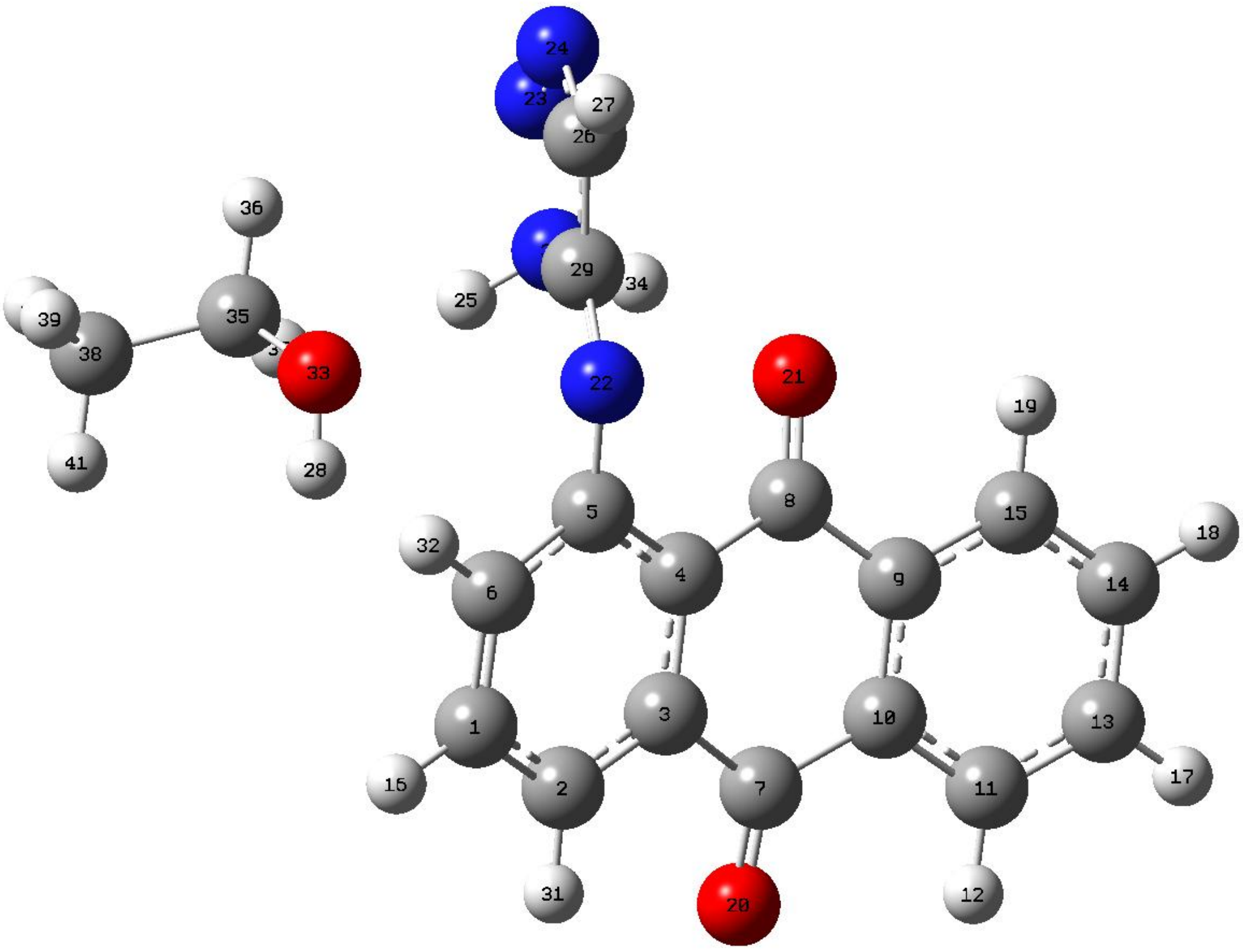

Center	Atomic	Atomic	Coordinates (Angstroms)		
Number	Number	Type	X	Y	Z
1	6	0	-0.210241	2.834116	-1.951533
2	6	0	0.967825	2.787852	-1.204352
3	6	0	1.391651	1.584326	-0.65856
4	6	0	0.621034	0.394884	-0.801275
5	6	0	-0.57812	0.441978	-1.5775
6	6	0	-0.94245	1.681664	-2.156297
7	6	0	2.707938	1.587751	0.050508
8	6	0	1.11267	-0.842281	-0.165711
9	6	0	2.528613	-0.894246	0.292908
10	6	0	3.294847	0.275556	0.4151
11	6	0	4.605933	0.204347	0.896094
12	1	0	5.178306	1.117941	0.99475
13	6	0	5.158333	-1.02577	1.234489
14	6	0	4.397556	-2.191966	1.110026
15	6	0	3.0856	-2.12584	0.653534
16	1	0	-0.540469	3.768079	-2.391185
17	1	0	6.178321	-1.079907	1.596218
18	1	0	4.828699	-3.150486	1.37397
19	1	0	2.482859	-3.020459	0.56648
20	8	0	3.296868	2.634841	0.289791
21	8	0	0.395542	-1.83271	0.008347
22	7	0	-1.369006	-0.625264	-1.969007

23	7	0	-3.284874	-2.399153	0.588598
24	7	0	-3.595316	-2.939334	-0.522013
25	1	0	-2.571178	-0.398183	0.608398
26	6	0	-2.989177	-2.435694	-1.609127
27	1	0	-3.183468	-2.812807	-2.598743
28	1	0	-2.610041	1.857162	1.174126
29	6	0	-2.123036	-1.379045	-1.255261
30	7	0	-2.256382	-1.321351	0.218636
31	1	0	1.574302	3.671829	-1.059719
32	1	0	-1.833896	1.698397	-2.771327
33	8	0	-3.18586	1.084167	1.217974
34	1	0	-1.331384	-1.583012	0.602063
35	6	0	-3.993388	1.155132	2.417257
36	1	0	-4.559795	0.223241	2.430635
37	1	0	-3.338242	1.16772	3.29375
38	6	0	-4.92111	2.357639	2.408842
39	1	0	-5.586774	2.330658	1.542911
40	1	0	-5.533212	2.359685	3.315186
41	1	0	-4.35426	3.29305	2.385216

7

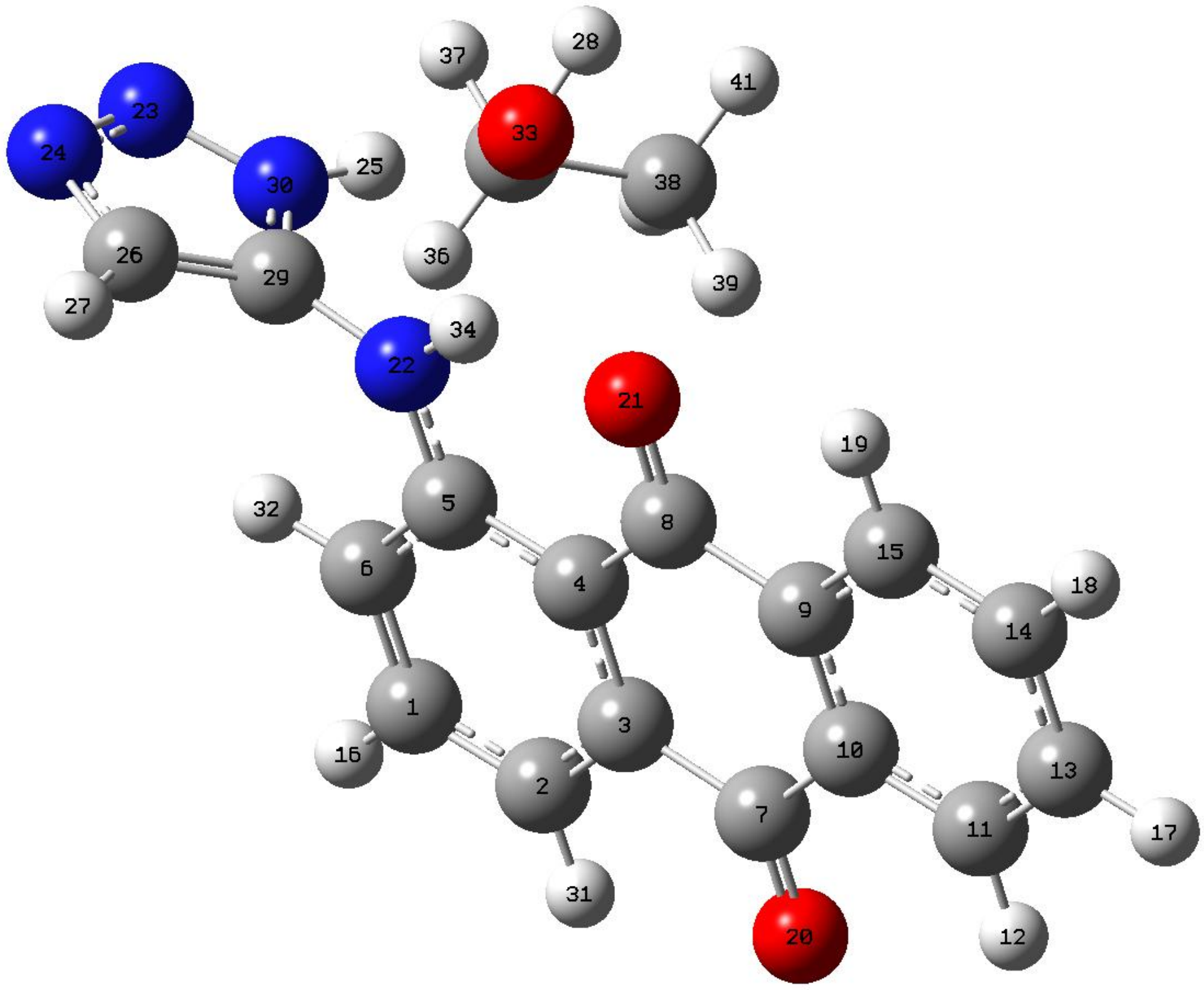

Center Number	Atomic Number	Atomic Type	Coordinates (Angstroms) X	Y	Z
1	6	0	0.570258	1.238797	2.545205
2	6	0	-0.783334	1.492696	2.305403
3	6	0	-1.454656	0.774097	1.326787
4	6	0	-0.787987	-0.218065	0.55837
5	6	0	0.597479	-0.45906	0.806526
6	6	0	1.250211	0.285427	1.814116
7	6	0	-2.900866	1.08289	1.112243
8	6	0	-1.522345	-0.977082	-0.461003
9	6	0	-2.962499	-0.6661	-0.68511
10	6	0	-3.625157	0.319241	0.065914
11	6	0	-4.978158	0.583755	-0.171322
12	1	0	-5.472181	1.346171	0.417406
13	6	0	-5.66894	-0.124231	-1.147852
14	6	0	-5.011024	-1.104999	-1.895355
15	6	0	-3.66639	-1.374442	-1.664861
16	1	0	1.095766	1.79282	3.314308
17	1	0	-6.716823	0.084296	-1.32865
18	1	0	-5.548791	-1.657562	-2.656867
19	1	0	-3.146828	-2.131928	-2.237037
20	8	0	-3.469724	1.940228	1.777627
21	8	0	-0.987205	-1.866427	-1.138564
22	7	0	1.287098	-1.401969	0.082518
23	7	0	4.837379	-1.48807	0.00318

24	7	0	4.626335	-2.603547	0.662121
25	1	0	3.586601	-0.058067	-0.796718
26	6	0	3.289123	-2.769298	0.830406
27	1	0	2.870417	-3.616532	1.348268
28	1	0	3.534137	1.340809	-2.690749
29	6	0	2.640385	-1.70419	0.240444
30	7	0	3.646895	-0.941618	-0.261512
31	1	0	-1.320172	2.240605	2.872719
32	1	0	2.296994	0.097723	2.015138
33	8	0	3.478487	1.438428	-1.732547
34	1	0	0.726698	-1.931524	-0.583335
35	6	0	4.152968	2.655618	-1.341952
36	1	0	4.099532	2.671521	-0.25244
37	1	0	5.20779	2.594436	-1.628303
38	6	0	3.497417	3.890445	-1.936747
39	1	0	2.450187	3.960906	-1.633129
40	1	0	4.017871	4.78948	-1.594474
41	1	0	3.543482	3.872452	-3.029701
